# Comparative Genomics Reveals Chd1 as a Determinant of Nucleosome Spacing *in Vivo*

**DOI:** 10.1534/g3.115.020271

**Published:** 2015-07-14

**Authors:** Amanda L. Hughes, Oliver J. Rando

**Affiliations:** Department of Biochemistry and Molecular Pharmacology, University of Massachusetts Medical School, Worcester, Massachusetts 01605

**Keywords:** chromatin, epigenomics, gene regulation

## Abstract

Packaging of genomic DNA into nucleosomes is nearly universally conserved in eukaryotes, and many features of the nucleosome landscape are quite conserved. Nonetheless, quantitative aspects of nucleosome packaging differ between species because, for example, the average length of linker DNA between nucleosomes can differ significantly even between closely related species. We recently showed that the difference in nucleosome spacing between two Hemiascomycete species—*Saccharomyces cerevisiae* and *Kluyveromyces lactis*—is established by *trans*-acting factors rather than being encoded in cis in the DNA sequence. Here, we generated several *S. cerevisiae* strains in which endogenous copies of candidate nucleosome spacing factors are deleted and replaced with the orthologous factors from *K. lactis*. We find no change in nucleosome spacing in such strains in which H1 or Isw1 complexes are swapped. In contrast, the *K. lactis* gene encoding the ATP-dependent remodeler Chd1 was found to direct longer internucleosomal spacing in *S. cerevisiae*, establishing that this remodeler is partially responsible for the relatively long internucleosomal spacing observed in *K. lactis*. By analyzing several chimeric proteins, we find that sequence differences that contribute to the spacing activity of this remodeler are dispersed throughout the coding sequence, but that the strongest spacing effect is linked to the understudied N-terminal end of Chd1. Taken together, our data find a role for sequence evolution of a chromatin remodeler in establishing quantitative aspects of the chromatin landscape in a species-specific manner.

Eukaryotic genomes are packaged into a repeating subunit, the nucleosome, comprising ∼147 bp of DNA wrapped around an octamer of histone proteins. Because the linker DNA between nucleosomes is more accessible to DNA-binding factors than is nucleosomal DNA, the detailed positions of nucleosomes across the genome are a subject of key importance for understanding transcriptional regulation (Zhang and Pugh 2011; Hughes and Rando 2014). Genome-wide surveys in a multitude of eukaryotes all reveal similar qualitative features of chromatin architecture, with nucleosome-depleted regions (NDRs) found at regulatory regions such as promoters and relatively well-positioned nucleosomes flanking these NDRs.

Multiple distinct mechanisms responsible for establishing the chromatin landscape have been identified over decades of study. Nucleosome assembly is thermodynamically disfavored over relatively stiff Poly(dA:dT) sequences (Drew and Travers 1985; Anderson and Widom 2001); therefore, in a number of species (such as the budding yeast *S. cerevisiae*) promoters are thought to “program” intrinsic nucleosome depletion using such sequences (Iyer and Struhl 1995; Sekinger *et al.* 2005; Yuan *et al.* 2005; Yuan and Liu 2008; Kaplan *et al.* 2009; Zhang *et al.* 2009). Although *cis*-acting genomic sequence plays a key role in establishing nucleosome depletion at yeast promoters, the vast majority of nucleosome positions are established by *trans*-acting factors, a fact emphasized by the vast improvement in recapitulating relatively accurate *in vivo* nucleosome positions by supplementing *in vitro* reconstitutions with whole cell extract (Zhang *et al.* 2011). The requirement for ATP in such reconstitution studies underlines the key role of the general class of ATP-dependent chromatin remodelers in establishing nucleosome positions *in vivo* (Clapier and Cairns 2009).

Although qualitative features of promoter chromatin architecture are conserved across species, quantitative features can differ significantly between even closely related species. We previously surveyed nucleosome positioning across 16 species of Ascomycete (Tsankov *et al.* 2010; Tsankov *et al.* 2011; Xu *et al.* 2012), finding that features such as average linker length differ dramatically between species in this phylogeny. These quantitative differences in chromatin architecture provide a mechanistic toolbox for understanding how chromatin structure is established. For example, using *Saccharomyces cerevisiae* strains (average linker ∼15–20 bp) carrying artificial chromosomes comprising large fragments of the *Kluyveromyces lactis* genome (average linker ∼30 bp), we found that nucleosomes over the *K. lactis* sequences adopted the shorter average linkers characteristic of *S. cerevisiae* (Hughes *et al.* 2012). This result demonstrates that nucleosome spacing is not encoded in the genomic sequence and instead is established by some *trans*-acting factor in the *S. cerevisiae* host environment.

Here, we sought to identify the “molecular ruler” responsible for the differences in nucleosome spacing between *K. lactis* and *S. cerevisiae*. We generated *S. cerevisiae* “factor swap” strains in which deletion of an endogenous gene was complemented with the *K. lactis* ortholog, and we performed MNase-Seq to map nucleosomes in these strains. We tested several candidates likely to play a role in establishing the global linker length in these organisms, finding no significant effect of interspecies differences in Isw1 or Hho1 on nucleosome spacing. In contrast, we confirmed that deletion of the ATP-dependent chromatin remodeler Chd1 causes a loss of 3′ nucleosome positioning (Gkikopoulos *et al.* 2011) and, more interestingly, found that *K. lactis* Chd1 not only was able to complement this loss of positioning but also generated nucleosomal arrays with increased spacing. Expression of chimeric Chd1 proteins revealed that sequences responsible for the increased linker length are dispersed throughout the protein, and that the greatest individual effect on linker length was observed for swaps affecting the understudied N-terminus of Chd1. Together, these results reveal that sequence differences in a single protein can drive substantial global changes in chromatin packaging between species.

## Materials and Methods

### Yeast strains

Yeast strains were derived from the diploid *CHD1* S288C strain, BY4743. The coding region of each gene was deleted with *URA3*, and URA+LYS+ (HIS+, for *chd1* strains undergoing integrative swap) haploid segregants were selected. *K. lactis HHO1* (Klla0D: 580781–582918) and *ISW1* (Klla0F: 645288–649248) genes flanked by *Xho*I and *Sac*II restriction sites were ligated into the pRS415 yeast centromeric plasmid with *LEU2* marker, whereas *IOC3* (Klla0F: 2162754–2159132) was cloned into pRS413 yeast centromeric plasmid with *HIS3* marker using *Xho*I and *Sac*I restriction digestion. The *S. cerevisiae* copy of *CHD1* (V:504762-509897) with engineered *Bam*HI and *Sac*II restriction sites was cloned into pRS415; the coding region was then replaced with that of *K. lactis* by recombination of the plasmid with a *K. lactis CHD1* PCR product with flanking *S. cerevisiae CHD1* sequence. The deletion strains were transformed with either vector alone or the plasmid bearing the *K. lactis* gene. For *CHD1*, the *K. lactis* complementation strains were also made via homologous recombination of the *K. lactis* PCR product into the *S. cerevisiae* endogenous locus in the haploid deletion strain. Transformants with this product were selected on 5-FOA media after an overnight outgrowth in YPD and integration was confirmed via colony PCR. Additionally, for a wild-type comparison, an *S. cerevisiae* PCR product was reintegrated into the deletion strain and selected as for the *K. lactis* complementation. Chimeric *CHD1* were generated first by recombination of portions of the *K. lactis* gene into the *S. cerevisiae* plasmid and then by transformation into the deletion strain, selecting for recombination into the endogenous locus by 5-FOA selection. PCR-based C-terminal tagging (Tagwerker *et al.* 2006) of Chd1 was performed: the HB module of pFA6-HBH-kanMX6 was replaced with HA sequence at PacI/AscI, and the resultant plasmid was used for generation of an HA-containing PCR product targeting the C-terminus of Chd1, which was integrated and selected for by kanamycin resistance.

### Yeast growth

Plasmid-bearing strains were grown in Synthetic Dextrose plus uracil media to maintain plasmids. Chd1 integrated strains were grown in YPD media. All yeasts were grown in 200 ml of media at 30° overnight until approximately 0.5 OD_600_.

### Mononucleosomal DNA isolation

Yeast cultures (200 ml) were fixed in 1.85% formaldehyde at 30° and shaking for 30 min. Cell pellets were resusupended in 1.2 ml of cell breaking buffer (10 mM Tris, pH 7.4, 20% glycerol) with Sigma protease inhibitors and split into two screw cap tubes with approximately 0.6 ml of 0.5 mm zirconium silica beads (Biospec Products). The cell wall was broken on a cold aluminum rack in a Mini Bead Beater (Biospec Products). The cells were collected and subjected to 20 min of micrococcal nuclease digestion (with a titration range of MNase levels, typically ∼1–20 Units) at 37° in NP buffer (50 mM sodium chloride, 10 mM Tris, pH 7., 5 mM magnesium chloride, 1 mM calcium chloride, 0.5 mM spermidine, 1 μl/ml β-mercaptoethanol, and 0.01% NP-40). The reaction was stopped (150 μl of 50% sodium dodecylsulfate, 0.05 M ethylenediaminetetraacetic acid) and proteins were removed with 5 μl 20 mg/ml proteinase K at 65° overnight. Digestions were chosen to have mostly mononucleosomal-sized DNA with some dinucleosomal and visible trinucleosomal DNA (Supporting Information, Figure S8)—the precise MNase amounts used for each condition differ based on the precise OD of cells collected and the strain being digested. Mononucleosomal DNA was purified by extraction with equal volume phenol:chloroform:isoamyl alcohol, precipitated with 0.1 volumes sodium acetate and 2.5 volumes ethanol, and treated with RNase solution (Sigma) at 37° for 1 hr. Digestion ladders were assessed by running 5 μl on a 2% agarose gel. Half (25 μl) of the appropriate digestion was treated with calf intestinal phosphatase (NEB) at 37° for 45 min. The mononucleosomal band was gel-purified from a 1.8% agarose gel using Freeze N’ Squeeze columns (BioRad), was extracted with an equal volume of phenol:chloroform:isoamyl alcohol, and was ethanol-precipitated overnight.

### Deep sequencing library preparation

Approximately 250 ng of mononucleosomal DNA was end-cleaned with END-it (Epicentre) in a 40 μl reaction. AMPure XP beads (Beackman Coulter) were used to clean the reaction with 1.8× beads and were washed once with 70% ethanol. The beads were resuspended with 21.25 μl water and the DNA was then A-tailed with Klenow exo- DNA Polymerase (Epicentre) in a 25 μl reaction on the beads. This reaction was cleaned on the beads by adding 1.8× ABR buffer (15% PEG, 2.5 M sodium chloride) and washed with 70% ethanol. The beads were resuspended with 11.75 μl water and 0.6 μl of 10 nM barcoded adapters was added; a 15 μl ligation reaction was performed at room temperature for 1 hr, the reaction mixture was made up to 25 μl, and the ligation was continued at 16° overnight. The ligation reaction was cleaned on the beads by adding 1.3× ABR buffer, washed twice with 70% ethanol, and eluted with 39 μl water. Two-thirds of the ligated DNA was used for two 25 μl Pfx PCR reactions using 10 and 12 cycles of the PCR extensions. A portion of the PCR reactions (4 μl) was run on a gel to check the presence and size of the product. Final PCR with the remaining one-third of the ligated material was repeated with the best cycling conditions for each sample. PCR reactions were pooled and mixed with 1.3× AMPure beads, washed on the beads two times with 70% ethanol, and eluted in 20 μl water. One-tenth of the purified library was used to quality-check the library by StrataCloning, which requires an initial A-tailing reaction with Taq DNA Polymerase. KAPA Library Quantification Kit was used on a serial dilution of 1 μl of each library to estimate the relative concentrations, and the libraries were mixed 1:1 and sent to the UMass deep sequencing core for paired-end Illumina Genome Analyzer IIx (*K.lac HHO1* [5652904 reads], *hho1d* [4950620 reads], *Klac IOC3ISW1* [4481125 reads], and *ioc3disw1d* [6429270 reads]), Illumina MiSeq (*Scer CHD1*plasmid [1248864 reads], *chd1d* [1482529 reads], *Scer CHD1* [1850101 reads], *Klac CHD1* [954556 reads], and *Klac CHD1*plasmid [1718781 reads]), and Illumina HiSequation 2000 (*Klac*Nterm [5738558 reads], *Klac*Cterm [5489420 reads], *Klac*N+Cterm [6008678 reads], *Klac*ATPase [6221156 reads], *ScerCHD1*plasmid #2 [11110533], *chd1d* #2 [12938481 reads], *ScerCHD1* #2 [16030049 reads], *KlacCHD1* #2 [9148367 reads], *KlacCHD1*plasmid #2 [14809596 reads], *Klac*N1-194 [31500245 reads], *Klac*N1-176 [27774619 reads], *Klac*N177-340 [36663544 reads], and *Klac*N195-340 [26284524 reads]).

### Data analysis

Raw fastq files were separated by barcode using Novobarcode. Sequences were aligned to the SacCer3 genome using bowtie2 with its defaults and filtered to keep only the uniquely mapped reads, separating reads into Watson and Crick alignment. To make TSS alignments, aligned reads were counted using genomeCoverageBed for every base pair. The cross-correlation between Watson and Crick reads was used to infer fragment length and reads were extended to the fragment length and recounted for the full fragment using genomeCoverageBed. The genome average base pair count was averaged to 1 and alignments were made using 500 bp upstream of the edge of the +1 nucleosome [as defined in Tsankov *et al.* (2010)] to the end of the gene, averaging normalized reads in 10 bp bins.

To call nucleosome positions, the first base pair of each read was added and the Watson and Crick reads were combined in order, first by chromosome and then by base pair, generating a tab delimited file with chromosome, base pair, F/R, and count. This file was then used in a Template Filtering algorithm with seven nucleosome templates [developed in Weiner *et al.* (2010)] to call nucleosome positions genome wide. To assign nucleosomes to genes and designate the +1 and −1 nucleosomes, a homemade perl script scanned nucleosome calls up to 500 bp upstream of the coding region to find the nucleosome depleted region (a linker length more than 100 bp) upstream of the gene, the +1 and −1 nucleosomes were designated as the flanking nucleosomes, and the positions of the genic nucleosomes were assigned to each gene. The average distance between the genic nucleosomes was determined for each gene, as was the distance between the center of the +1 and +3 and +3 and +5 nucleosomes. The distance of each genic nucleosome from the edge of the +1 nucleosome was determined; only nucleosomes that shifted less than 150 bp were considered (to avoid cases in which nucleosomes were lost/gained or miscalled) to calculate average shifts and obtain p-values using a two-tailed, paired *t*-test.

The RNA Polymerase II occupancies defined in the unstressed wild-type strain in Kim *et al.* (2010) were used to separate genes by Pol2 occupancy. The wild-type and *chd1* mutant strain turnover rates from Radman-Livaja *et al.* (2012) were used to separate genes by average coding region turnover as well as the difference in 3′ turnover.

### Data availability

Data are available at GEO, accession #GSE66979.

## Results

We set out to identify the molecular basis for the difference in average nucleosome spacing between *Kluyveromyces lactis*, (∼175–180 bp repeat length) and *Saccharomyces cerevisiae* (∼160–165 bp repeat length) (Heus *et al.* 1993; Tsankov *et al.* 2010). Because traditional QTL mapping cannot be performed between reproductively isolated species, we instead took a candidate approach to the question of how quantitative differences in nucleosome spacing arise. To test specific chromatin remodelers for internucleosomal spacing function, we deleted candidate factors from *S. cerevisiae* and complemented them with the orthologous gene from *K. lactis*. Depending on the number of loci contributing to this quantitative trait, mapping nucleosomes genome-wide in the deletion and complementation strains could potentially identify key factors underlying this evolutionary divergence in nucleosome spacing.

Our initial list of factors included the linker histone H1 (encoded by *HHO1*) and several ATP-dependent remodeling enzymes including Isw1-containing complexes and the Chd1 remodeler. H1 has long been understood to play a role in nucleosome spacing (Van Holde 1989) because, for example, nucleosome spacing significantly decreases in murine ES cells lacking three of the five H1 isoforms encoded in the mouse genome (Fan *et al.* 2005). Moreover, a recent physical modeling study found that nucleosome spacing in 11 out of 12 fungal species could be modeled with a two parameter model, with the sole failure of the model—to explain the long spacing of *K. lactis*—being hypothesized to reflect H1 in this species interfering with DNA “breathing” (Mobius *et al.* 2013). The rationale for choosing Isw1 and Chd1 ATP-dependent remodelers is based on the well-established role for Isw-class remodelers in nucleosome sliding and spacing *in vitro* (Ito *et al.* 1997; Vary *et al.* 2003; Stockdale *et al.* 2006; Yang *et al.* 2006), as well as the dramatic loss of periodic nucleosome spacing observed in yeast lacking Isw1 and Chd1 (Gkikopoulos *et al.* 2011). Additionally, the structure of ISW1a DNA binding domains in complex with nucleosomes has suggested a mechanism for physically measuring linker length (Yamada *et al.* 2011).

**Figure 1:**
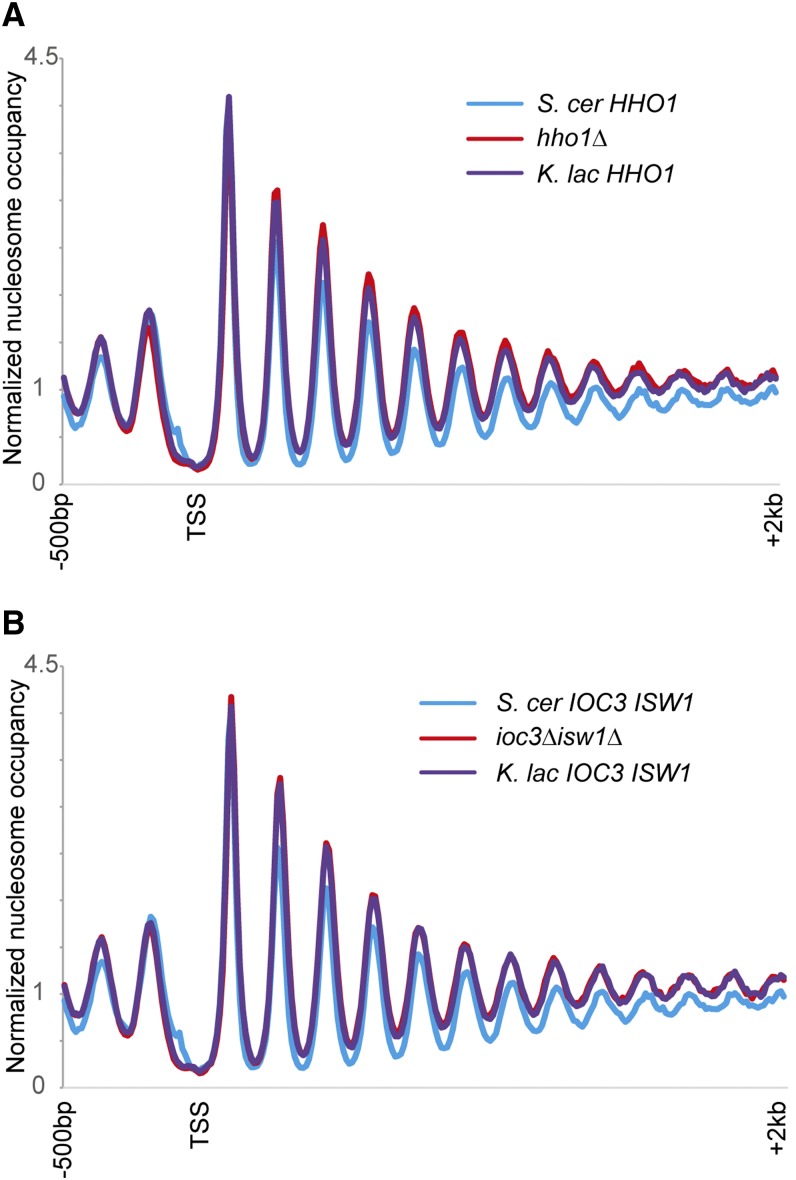
Isw1a and Hho1 are not responsible for nucleosome spacing differences between *S. cerevisiae* and *K. lactis*. (A) Genome-wide nucleosome mapping data are shown for all *S. cerevisiae* genes, aligned by the transcription start site (TSS). Data are shown for wild-type yeast, yeast lacking the *HHO1* gene, and *hho1*Δ yeast expressing the *HHO1* ortholog from *K. lactis*. (B) As in (A), but for double mutations in *ISW1* and *IOC3*. For all nucleosome mapping data in this figure and below, the y-axis shows normalized nucleosome occupancy, with a value of 1 being the genome-wide average occupancy of MNase-Seq reads. Note that data for BY4741 here are from an underdigested MNase sample, resulting in differences in nucleosome occupancy, but no effect on linker lengths.

Genome-wide nucleosome mapping in *S. cerevisiae* strains lacking these factors, or carrying the *K. lactis* orthologs, revealed no effect of histone H1 or of the Isw1a complex in establishment of nucleosome spacing *in vivo* (Figure 1). For Isw1, we generated deletion and swap strains for the ATPase subunit Isw1 in combination with Ioc3 (which binds Isw1 to form the Isw1a complex). Deletion of either the Isw1a complex or histone H1 does not have a general effect on chromatin structure, particularly with regard to internucleosomal spacing. Swap strains carrying the *K. lactis* orthologs of these factors do not exhibit increased nucleosome spacing as assayed by genome-wide nucleosome mapping, suggesting that sequence differences in these proteins are not responsible for the difference in average linker length between these species (or that the *K. lactis* orthologs are not functional in *S. cerevisiae*; see *Discussion*).

**Figure 2:**
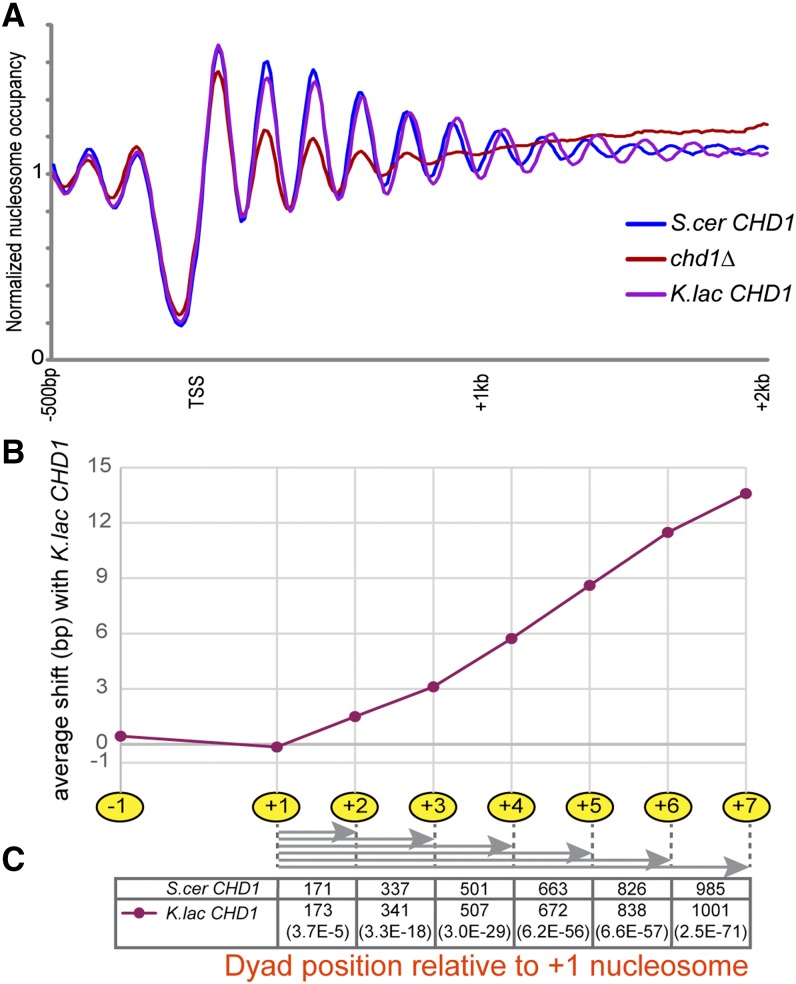
The *K. lactis CHD1* ortholog can direct longer average linkers in *S*. *cerevisiae*. (A) Nucleosome mapping for *chd1*Δ and *CHD1* swap strains, as in Figure 1. (B) Quantitation of the shift in average nucleosome position for *CHD1* swap strains. Nucleosome positions were called as in Weiner *et al.* (2010), and the average distance between nucleosomes in the *K. lactis* swap strain and the same nucleosomes in the strain with *S. cerevisiae CHD1* is plotted for nucleosome positions −1 to +7. (C) Distance of each coding region nucleosome from the +1 nucleosome, for the *S. cer* and *K. lac CHD1*-containing strains, with corresponding p-values (paired, two-tailed *t*-test) shown in parentheses at the bottom.

In contrast, deletion of *CHD1* alone dramatically alters the global chromatin in *S. cerevisiae*, particularly for nucleosomes over the middle and 3′ ends of coding regions, consistent with published reports (Gkikopoulos *et al.* 2011) (Figure 2A, Figure S1). Interestingly, complementation of this deletion with *K. lactis CHD1* causes an increase in internucleosome spacing at the 3′ end of genes. This was observed in multiple biological replicates, and in strains where the *K. lactis* gene was either carried on a plasmid or integrated into the *S. cerevisiae* genome (Figure S2). By calling nucleosome peaks and determining the distance between adjacent nucleosomes, we see that nucleosomes throughout coding regions are spaced farther apart in the presence of *K. lactis* Chd1, although this effect is somewhat stronger for nucleosomes within the gene body than for 5′ nucleosomes (Figures 2, B and C, Figure S3). Although the nucleosome repeat length increases by ∼1.5 bp in the presence of *K. lactis* Chd1 at the 5′ end, *K. lactis* Chd1 expands the average internucleosome spacing by 3 bp subsequent to the +3 nucleosome (Figures 2, B and C, Figure S2, Figure S3). Chd1 thus appears to be involved in the measurement of internucleosome distances, and evolutionary divergence between the *S. cerevisiae* and *K. lactis* copies results in altered measurement of linker length (see *Discussion*). It is important to note that other factors must play a role in establishing linker length, because the increase in nucleosome spacing seen with the introduction of *K. lactis* Chd1 does not fully account for the ∼15 bp longer average linkers seen in the *K. lactis* genome.

**Figure 3:**
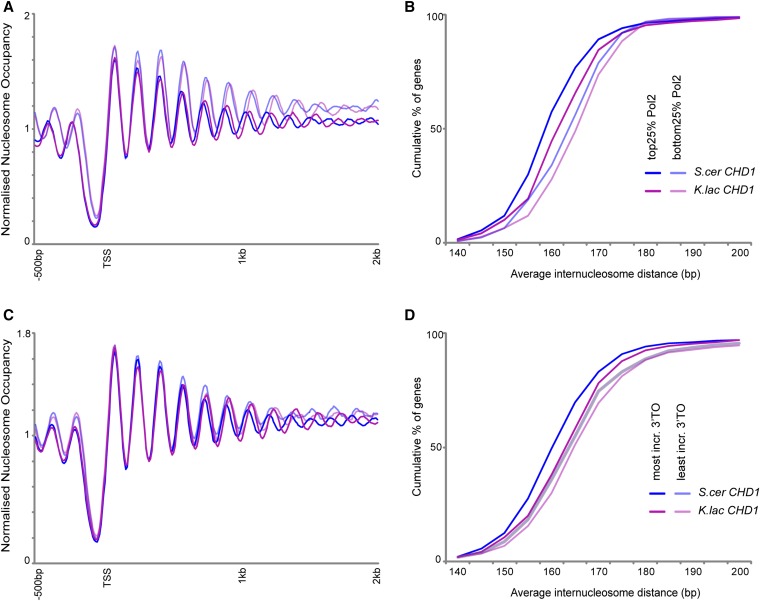
Chd1 swaps preferentially affect highly transcribed genes. Nucleosome mapping (A) and cumulative distribution plots (B) for *chd1*Δ and *CHD1* swap strains, with data shown for the 25% most Pol2-enriched or Pol2-depleted genes (Kim *et al.* 2010), as indicated in (B). These plots recapitulate known effects of transcription on genic chromatin (Tsankov *et al.* 2010; Weiner *et al.* 2010; Valouev *et al.* 2011), with lower nucleosome occupancy but tighter internucleosome spacing readily observed in comparing these gene sets for wild-type. (C and D) As in (A and B), but for genes broken into categories according to previously reported effects of Chd1 on replication-independent histone turnover (Radman-Livaja *et al.* 2012).

The analyses in Figure 2 provide a global view of average nucleosome spacing across the genome. Such ensemble measurements of course potentially obscure gene-specific variation in nucleosome spacing driven by the Chd1 swap—it is possible to infer information about the regulation and targeting of Chd1 activity by determining where the greatest effects of Chd1 are found. Chd1 genetically and physically interacts with yFACT as well as the Paf1 complex (Simic *et al.* 2003; Biswas *et al.* 2007), and it has been hypothesized that Chd1 exerts its effects on gene body nucleosomes by binding to the longer extranucleosomal DNA present as elongating RNA polymerase exposes nucleosomal DNA (Zentner *et al.* 2013). If the function of Chd1 is targeted to gene body nucleosomes via RNA polymerase elongation, then the increased linker length in the presence of *K. lactis* Chd1 should be stronger at more highly transcribed genes. Separating genes into quartiles based on RNA Polymerase II occupancy in their coding regions (Kim *et al.* 2010) revealed that highly expressed genes exhibit a greater increase in nucleosome spacing in the presence of *K. lactis* Chd1 than the lowest expressed genes (Figure 3, A and B), consistent with a potentially cotranscriptional role for Chd1 in nucleosome spacing. In addition, two previous studies identified a role for Chd1 in suppressing replication-independent histone turnover at the 3′ ends of genes, particularly at relatively long coding regions (Radman-Livaja *et al.* 2012; Smolle *et al.* 2012). Interestingly, those genes that show the greatest increase in 3′ turnover in *chd1*Δ mutants also exhibit a greater increase in spacing in strains carrying the *K. lactis* Chd1 (Figure 3, C and D)—these genes are thus the most responsive to both primary activities of Chd1 *in vivo*. Moreover, our finding of greater effects of the Chd1 swap on mid-coding region nucleosomes is consistent with previous reports of Chd1 association with the mid-gene histone mark H3K36me3 (Smolle *et al.* 2012).

**Figure 4:**
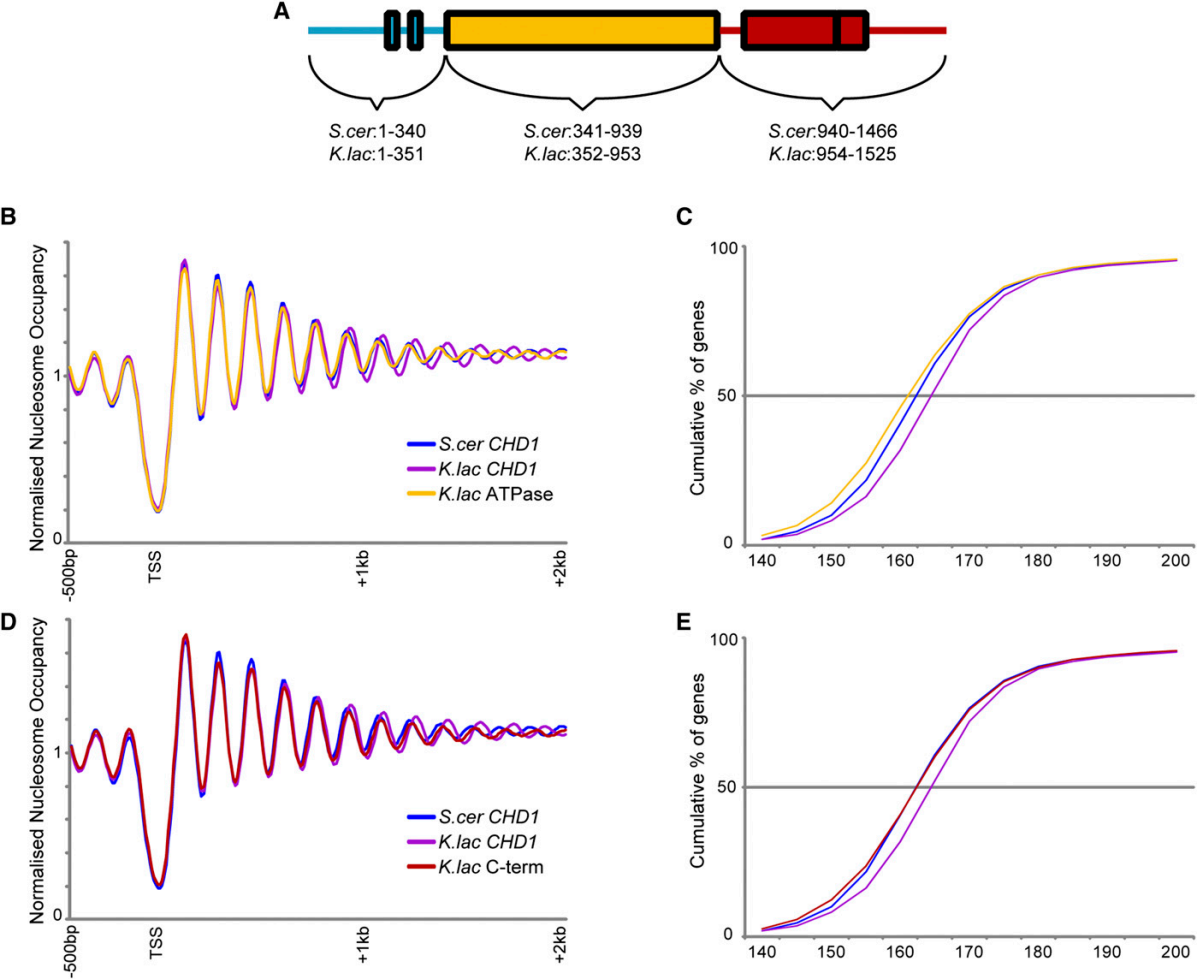
Minimal effects of Chd1 ATPase and C-terminal domains on nucleosome spacing. (A) Schematic of Chd1 protein domains. (B and C) Genome-wide nucleosome mapping data (B) and cumulative distribution plot of internucleosome distance (C) for strains carrying *CHD1* from *S. cerevisiae*, *K. lactis*, or a chimeric *CHD1* with the *K. lactis* ATPase domain in the context of the *S. cerevisiae CHD1* sequence. (D and E) As in (B and C), but for swaps affecting the Chd1 C-terminus.

Having identified a pair of Chd1 orthologs that direct different internucleosome spacing in an otherwise identical nuclear environment, we sought to generate chimeric Chd1 proteins to identify the “molecular ruler” responsible for linker length. ATP-dependent chromatin remodelers bear a similar ATPase motor but have distinct accessory domains/subunits that recognize DNA and histone modifications and variants (Clapier and Cairns 2009). These domains regulate the localization and activity of the remodelers. In Chd1, these accessory domains include tandem chromodomains at the N-terminus and DNA binding domains at the C-terminus that flank the central helicase. To test which domain(s) of Chd1 are responsible for its measurement activity, we generated chimeras between *K. lactis* and *S. cerevisiae* Chd1 (Figure 4A), and we tested these chimeric proteins for longer measurement in the *S. cerevisiae* genome.

**Figure 5:**
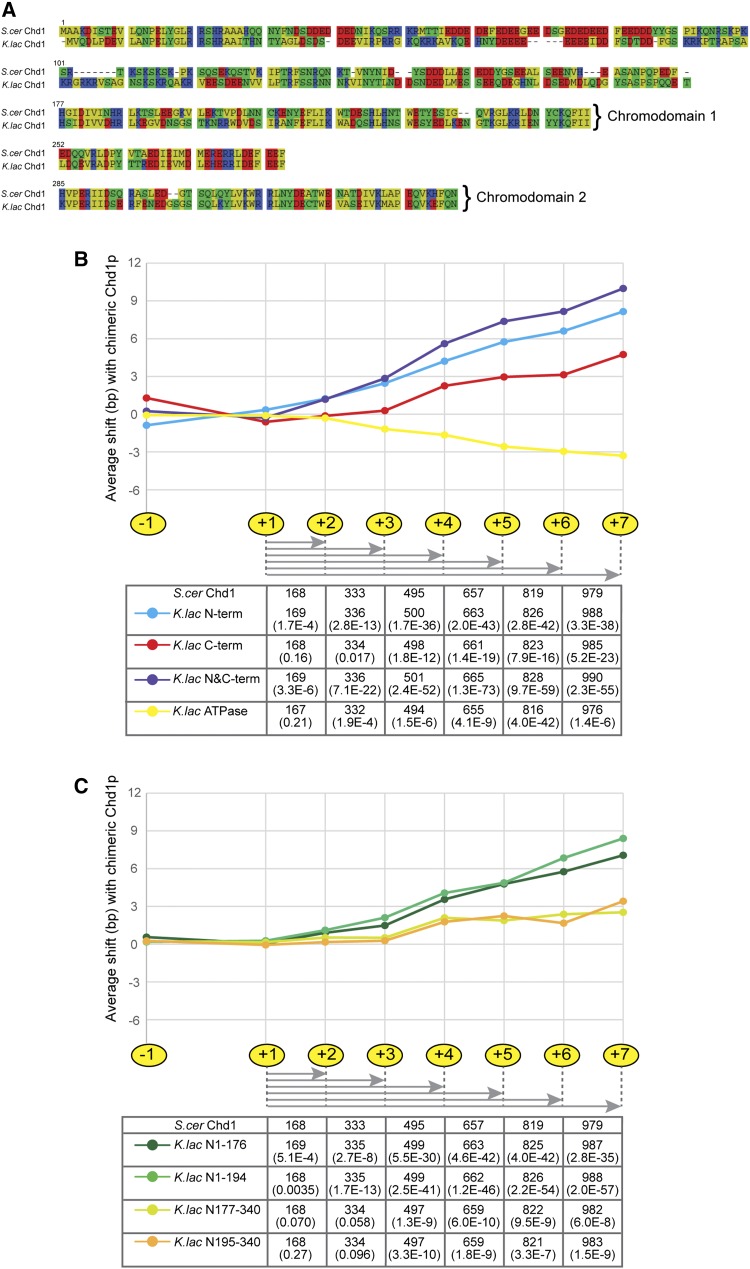
N-terminus of Chd1 has the greatest effects on nucleosome spacing. (A) Alignment of the N-terminal protein sequences for the two orthologs of Chd1, generated in SeaView version 4 (Gouy *et al.* 2010): nonpolar residues in yellow; polar, uncharged in green; basic in blue; and acidic in red. (B and C) Shifts in nucleosome position as compared to the *S. cer CHD1* strains are plotted for nucleosome calls −1 to +7; the distances from the +1 nucleosome are indicated in the table for the *S. cer* and various chimeric *CHD1* strains, with corresponding p-values.

As expected, swapping the highly conserved ATPase domains between Chd1 orthologs did not generate any increase in linker measurement (Figures 4, B and C). Replacing the C-terminus of *S. cerevisiae* Chd1 with that from *K. lactis* caused a very slight increase in nucleosome spacing (Figures 4, D and E). Overall, the greatest effect of a single domain swap was observed in chimeric proteins carrying the N-terminus of Chd1 from *K. lactis* (Figure S4), although this mutant did not recapitulate the full effect of the full protein swap, indicating that the sequences responsible for the long internucleosomal spacing directed by the *K. lactis* ortholog of Chd1 are distributed throughout the coding sequence. Indeed, swaps carrying both the N-terminal and C-terminal domains of the *K. lactis* protein exhibited the greatest effect on spacing of any tested chimaeras (Figure 5, Figure S4).

 Because the N-terminal domain of Chd1 had the greatest, albeit subtle, impact on nucleosome spacing, we finally sought to further narrow the region of the N-terminus that confers internucleosome spacing activity on Chd1. This N-terminal domain includes two chromodomains as well as an extreme N-terminal region, of which a large portion (residues 1–117) is typically truncated in proteins used for biochemical analyses and which is thus of unknown function. The N-terminal chromodomains have been shown to regulate the activity of Chd1 by interfering with the association of the ATPase lobes; it has been proposed that histone binding induces a conformational change that allows DNA binding by the helicase domain and ATP hydrolysis (Hauk *et al.* 2010). Comparing the sequences of the two chromodomains of Chd1 between *S. cerevisiae* and *K. lactis* reveals that chromodomain 2 (residues 285–339) is highly conserved, and chromodomain 1 (residues 177–251) is slightly more divergent (fifth and third line, respectively, of Figure 5A). To test the involvement of the chromodomains and extreme N-terminus, we generated chimerae for the N-terminus. Surprisingly, we see that swaps affecting the extreme N-terminal portion of Chd1, comprising amino acids 1–176, led to a greater increase in average genic nucleosome spacing than did swaps targeting the two chromodomains (residues 177–339) (Figure 5, B and C, Figure S5, Figure S6).

Taken together, these results show that the difference in spacing activity between Chd1 orthologs from *K. lactis* and *S. cerevisiae* is distributed throughout the protein, because the full-length Chd1 has a greater effect than any chimera on nucleosome spacing, but that the N-terminal portion of Chd1 contributes more to this difference in spacing than does any other individual region of Chd1 tested. This suggests that the Chd1 proteins of these species do not differ in a single domain that acts as a physical ruler but rather differ in the contributions of multiple domains to the modulation of enzyme activity, conformation, localization, or stability.

## Discussion

Here, we investigate the molecular basis underlying the difference in average linker length between two Hemiascomycetous species, *K. lactis* and *S. cerevisiae*. By swapping orthologous genes from *K. lactis* into *S. cerevisiae*, we find that sequence differences in the ATP-dependent chromatin remodeler Chd1 can impact average internucleosome distance in an otherwise constant genetic background. Further dissection of the sequences responsible for this difference in internucleosome spacing reveals that nucleosome spacing activity is distributed throughout the Chd1 sequence, but surprisingly finds that the greatest effects on linker length can be attributed to the extreme N-terminus of this protein. Taken together, our results do not find a single “molecular ruler” responsible for an organism’s average internucleosome spacing, instead supporting a model in which many contributors to nucleosome spacing are distributed throughout multiple relevant proteins.

Interestingly, despite substantial evidence that histone H1 (Fan *et al.* 2005) and Isw1a (Vary *et al.* 2003; Bouazoune *et al.* 2009) could play a role in altering average linker length, swap strains affecting these factors revealed no difference between *S. cerevisiae* and *K. lactis* orthologs on internucleosome spacing. Although this suggests that sequence divergence in these factors is not responsible for interspecies differences in nucleosome spacing, it must be noted that this result could also reflect a lack of function of the introduced factors outside of their native genomic context. For example, the Isw1a complex from *K. lactis* may only be functional in the presence of the *K. lactis* ortholog of some recruiting transcription factor due to divergence in the TF-Isw1a binding interfaces between these two species. In other words, a negative result in this system is not definitive proof that Isw1a or histone H1 does not contribute to the measurement of internucleosome spacing. That said, the lack of nucleosome spacing phenotype in the deletion mutants affecting these factors also supports the hypothesis that these chromatin regulators do not play a central role in establishing global linker lengths *in vivo*.

In contrast, our results find a significant role for sequence differences between two Chd1 orthologs in directing different average linker length between these two species. These results are consistent with prior *in vivo* studies showing a key role for Chd1 in establishing phased arrays of nucleosomes (Gkikopoulos *et al.* 2011; Pointner *et al.* 2012), a result that we replicate, and with *in vitro* studies on the nucleosome centering and spacing activities of Chd1 (Stockdale *et al.* 2006; Pointner *et al.* 2012). The effect of the Chd1 swap is greatest at the most highly expressed genes, providing additional evidence supporting the notion that Chd1 function is targeted to transcribed regions, either via interactions with elongation-associated factors or as a result of binding to transiently increased extranucleosomal DNA from transcription-associated histone turnover or breathing.

Finally, the difference in nucleosome spacing activity observed between the two Chd1 orthologs raised the question of how linker length is “measured” by the Chd1 protein. Domain swap experiments reveal that the difference in spacing activity is distributed throughout the coding sequence, arguing against a “molecular ruler” such as an alpha helix whose length dictates DNA linker length. Surprisingly, although the spacing activity of Chd1 is broadly distributed, the N-terminus of Chd1 appears to direct most of the increased measurement seen in *K. lactis*. This activity is primarily localized to the understudied region N-terminal to the chromodomains, a region that has not been structurally characterized and is generally deleted in biochemical assays. The mechanistic basis by which this domain alters internucleosome spacing will be of interest—possibilities include effects on Chd1 folding, ATPase activity, recruitment, or protein stability or levels, although this last possibility is unlikely given the similar protein levels we measure here (Figure S7). In addition, because the differences we measure here are similar in magnitude to those previously reported for *yta7*Δ and *rtt106*Δ yeast (Lombardi *et al.* 2015), it will be interesting to determine whether Yta7 or Rtt106 recruitment or activity is affected by the sequence of the Chd1 protein. At present, based on the distributed effects of sequence differences on quantitative linker length, we speculate that sequence differences between species could affect the overall activity of the Chd1 remodeler, but this hypothesis will be challenging to address in an *in vivo* context. Nonetheless, given the prominent role for the understudied N-terminus in nucleosome spacing uncovered here, this domain may be of interest in biochemical investigations of Chd1 in the future.

## Supplementary Material

Supporting Information
