# Supplementary material for: Comparative Genomics Reveals Chd1 as a Determinant of Nucleosome Spacing in Vivo
Source: G3 (Bethesda). 2015 Jul 14;5(9):1889–97. doi: 10.1534/g3.115.020271 (PMC4555225; doi:10.1534/g3.115.020271)
Supplement: Supporting Information [file supp_5_9_1889__index.html]

Comparative Genomics Reveals Chd1 as a Determinant of Nucleosome Spacing in Vivo — Supporting Information 

# Comparative Genomics Reveals Chd1 as a Determinant of Nucleosome Spacing *in Vivo*

## Supporting Information for Hughes and Rando, 2015

**Files in this Data Supplement:**

- Supporting Information - Figures S1-S8 (PDF, 1 MB)
- Figure S1 - Individual gene examples showing effects of *K. lactis CHD1* orthologue on nucleosome positioning. (PDF, 331 KB)
- Figure S2 - The *K. lactis CHD1* orthologue can direct wider spacing of *S. cerevisiae* nucleosomes from both plasmid and endogenous expression. (PDF, 263 KB)
- Figure S3 - Distribution of nucleosome spacing changes in *CHD1* swap strains. (PDF, 200 KB)
- Figure S4 - Differences between *S. cerevisiae* and *K. lactis* Chd1 that affect nucleosome spacing are distributed throughout the protein. (PDF, 238 KB)
- Figure S5 - Genome-wide data for N-terminal swaps. (PDF, 229 KB)
- Figure S6 - Distribution of nucleosome position changes in N-terminal swaps. (PDF, 141 KB)
- Figure S7 - Chd1 abundance is unaffected by *K. lactis* sequence. (PDF, 200 KB)
- Figure S8 - Example MNase digestions. (PDF, 552 KB)
