# Supplementary material for: Comparative Genomics Reveals Chd1 as a Determinant of Nucleosome Spacing in Vivo
Source: G3 (Bethesda). 2015 Jul 14;5(9):1889–97. doi: 10.1534/g3.115.020271 (PMC4555225; doi:10.1534/g3.115.020271)
Supplement: Supporting Information [file supp_g3.115.020271_020271SI.pdf]

G3 · Genes | Genomes | Genetics

Comparative genomics reveals Chd1 as a determinant of nucleosome spacing in vivo

Amanda L. Hughes[1] and Oliver J. Rando[1†]

[1]*Department of Biochemistry and Molecular Pharmacology, University of Massachusetts Medical School, Worcester, MA 01605, USA*

† To whom correspondence should be addressed. Email: Oliver.Rando@umassmed.edu

DOI: 10.1534/g3.115.020271

Figure S1

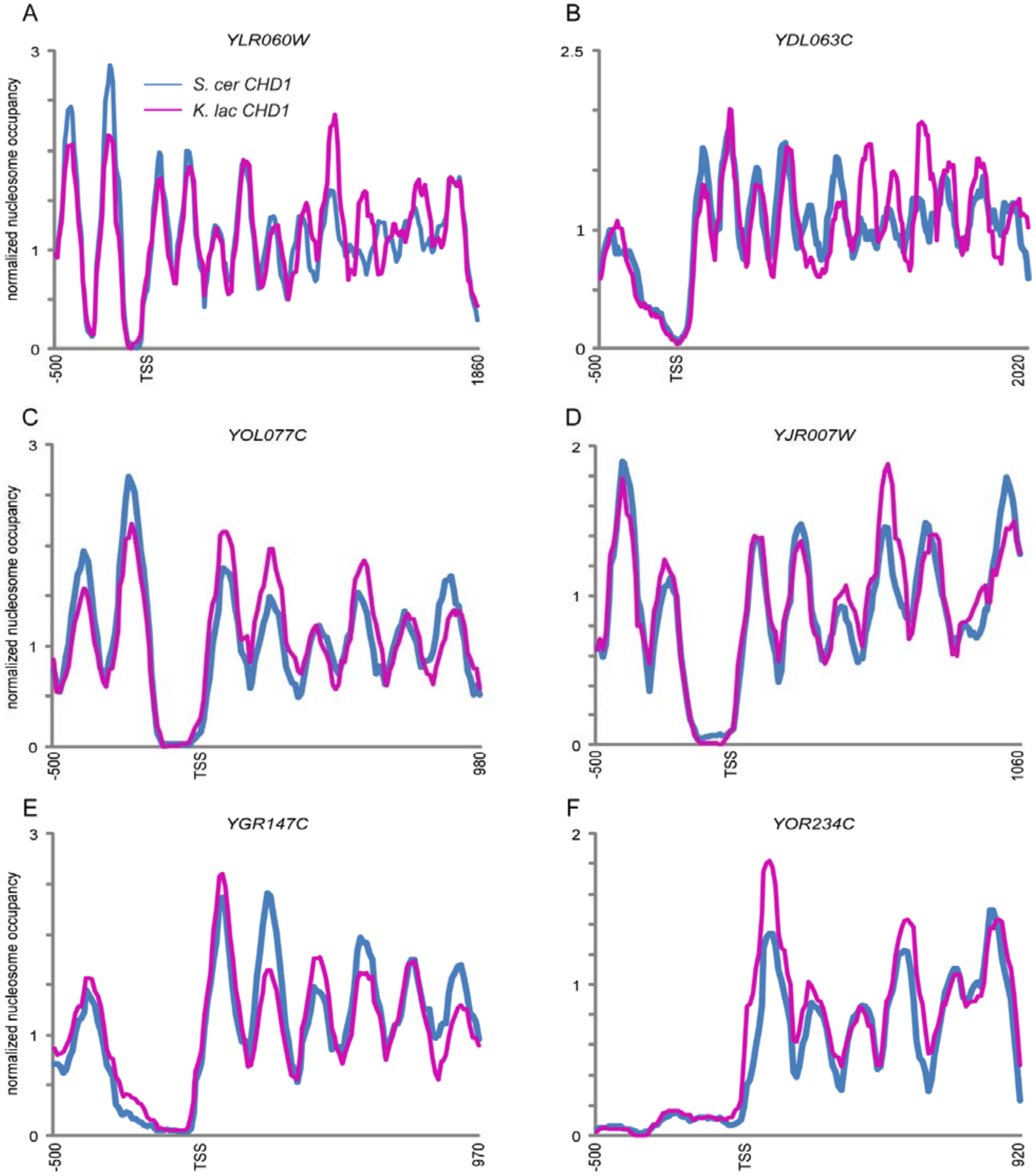

Figure S1 Individual gene examples showing effects of *K. lactis CHD1* orthologue on nucleosome positioning. Six individual genes from the dataset shown in **Figure 2**, including examples with evident nucleosome shifts as well as genes with no discernable effects of the *CHD1* swap.

Figure S2

A

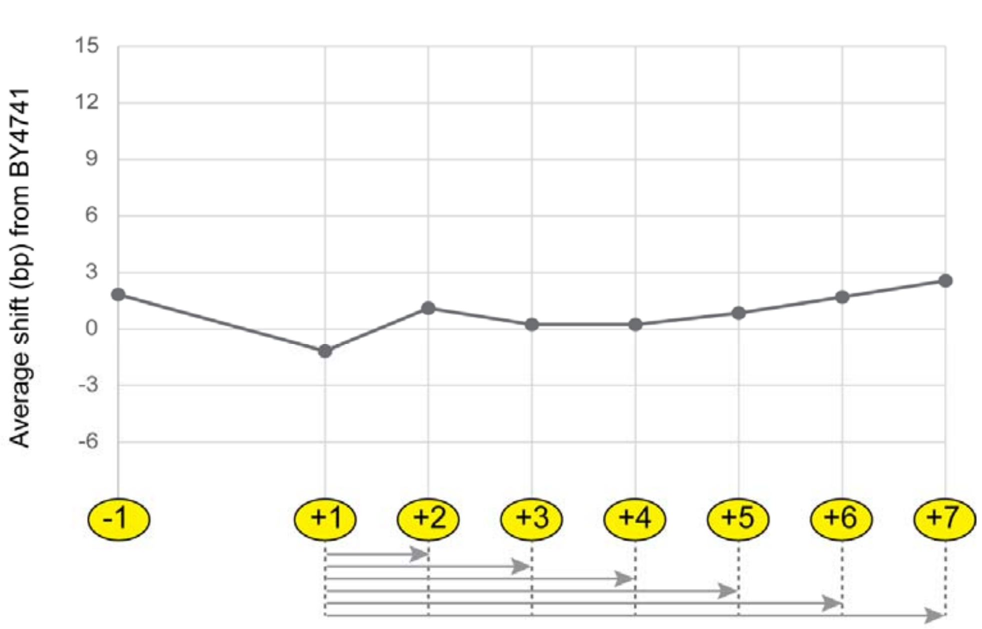

BY4741	168	334	495	655	815	971
S.cer Chd1 (integrated)	168<br>(0.25)	333<br>(0.96)	495<br>(0.52)	656<br>(0.26)	816<br>(0.31)	972<br>(0.89)

B

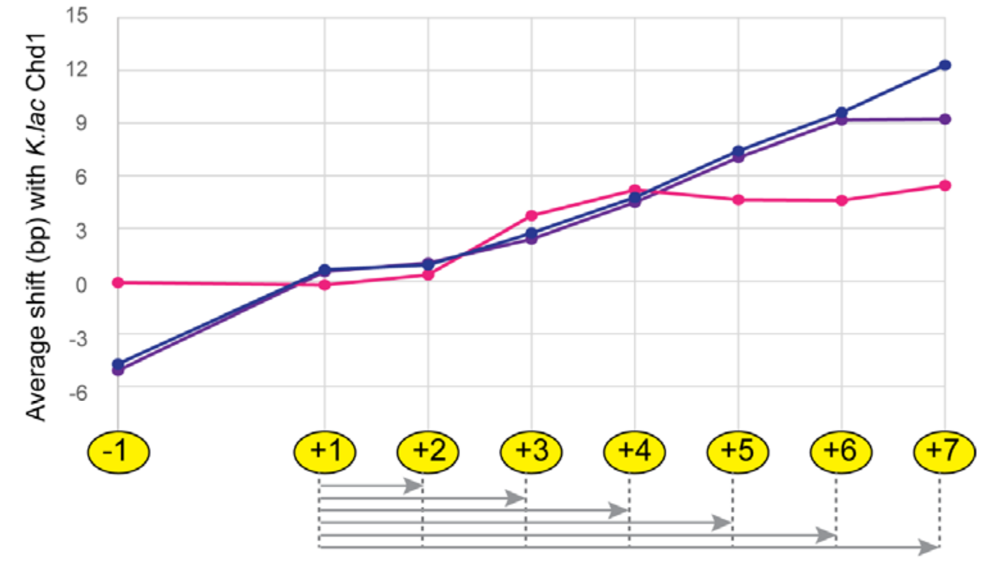

S.cer Chd1 (integrated)2	169	332	495	658	819	975
K.lac Chd1 (integrated)2	170<br>(0.069)	336<br>(8.8E-7)	502<br>(1.8E-12)	665<br>(5.8E-11)	825<br>(2.3E-8)	983<br>(1.9E-7)
S.cer Chd1 (plasmid)	170	336	501	666	828	991
K.lac Chd1 (plasmid)	175<br>(2.2E-7)	344<br>(6.3E-12)	512<br>(2.8E-15)	679<br>(1.2E-17)	843<br>(2.7E-19)	1007<br>(8.9E-15)
S.cer Chd1 (plasmid)2	172	338	505	669	833	999
K.lac Chd1 (plasmid)2	177<br>(1.1E-7)	345<br>(8.5E-14)	514<br>(3.8E-16)	682<br>(5.3E-23)	849<br>(9.0E-29)	1019<br>(4.0E-30)

Figure S2 The *K. lactis CHD1* orthologue can direct wider spacing of *S. cerevisiae* nucleosomes from both plasmid and endogenous expression. Plot of shifts in -1 to +7 nucleosome positions between *S. cerevisiae* and *K. lactis CHD1* swap strains, as in **Figure 2B-C**. In each case, top panel shows distances between equivalent nucleosomes for the pair of strains indicated, while bottom panel shows the average distance of genic nucleosomes from the +1 nucleosome, along with corresponding p-values for the swap strains. (**A**) shows that this metric is specific, as no significant changes are found between wild-type yeast and a "pseudo wild-type" with a genomic *chd1Δ* deletion covered by a plasmid-borne *CHD1* gene. (**B**) shows that effects of *K. lactis CHD1* on nucleosome spacing are reproducible both for genomically-integrated swap strains as well as plasmid-borne *CHD1* swaps.

Figure S3

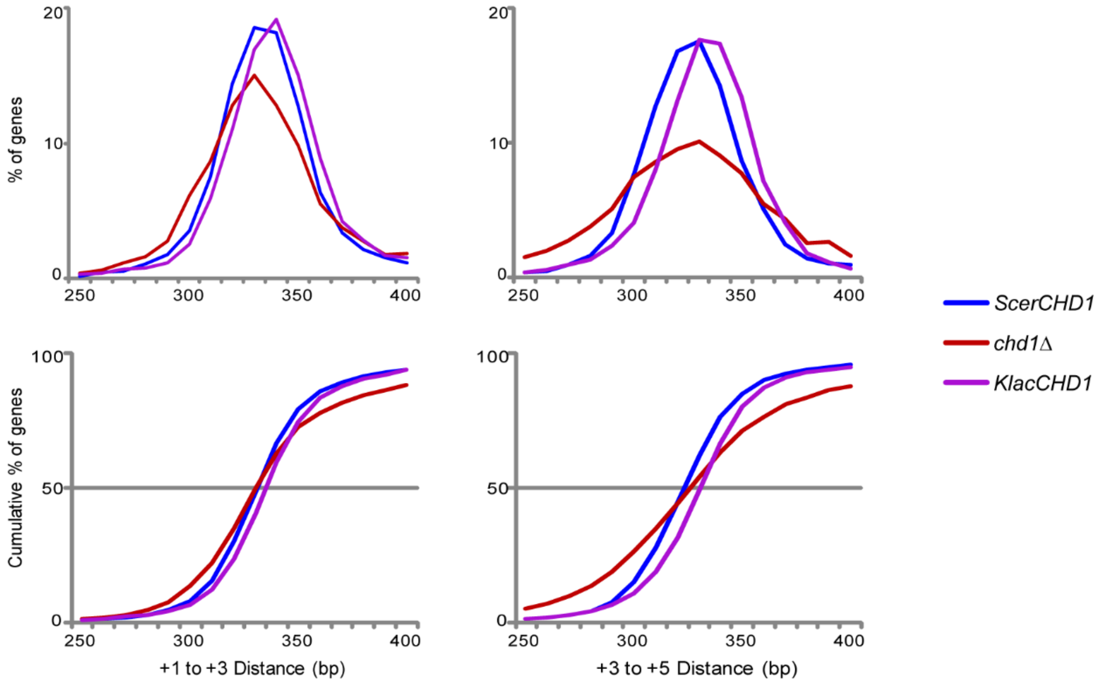

Figure S3 Distribution of nucleosome spacing changes in *CHD1* swap strains. Quantitation of internucleosome distances in the three strains detailed in **Figure 2A**. For each nucleosome mapping dataset, nucleosome positions were called as in (WEINER *et al.* 2010). Left panels show distances from all ORF +1 nucleosomes to the corresponding +3 nucleosome, while right panels show +3 to +5 nucleosome distances. Top panels show the histogram of all such distances for the three strains, color-coded as in (A), while bottom panels show the same data as cumulative distribution plots. A replicate for these integrated strains as well as 2 replicates for plasmid-borne *CHD1* strains are shown in **Supplemental Figure S2**.

Figure S4

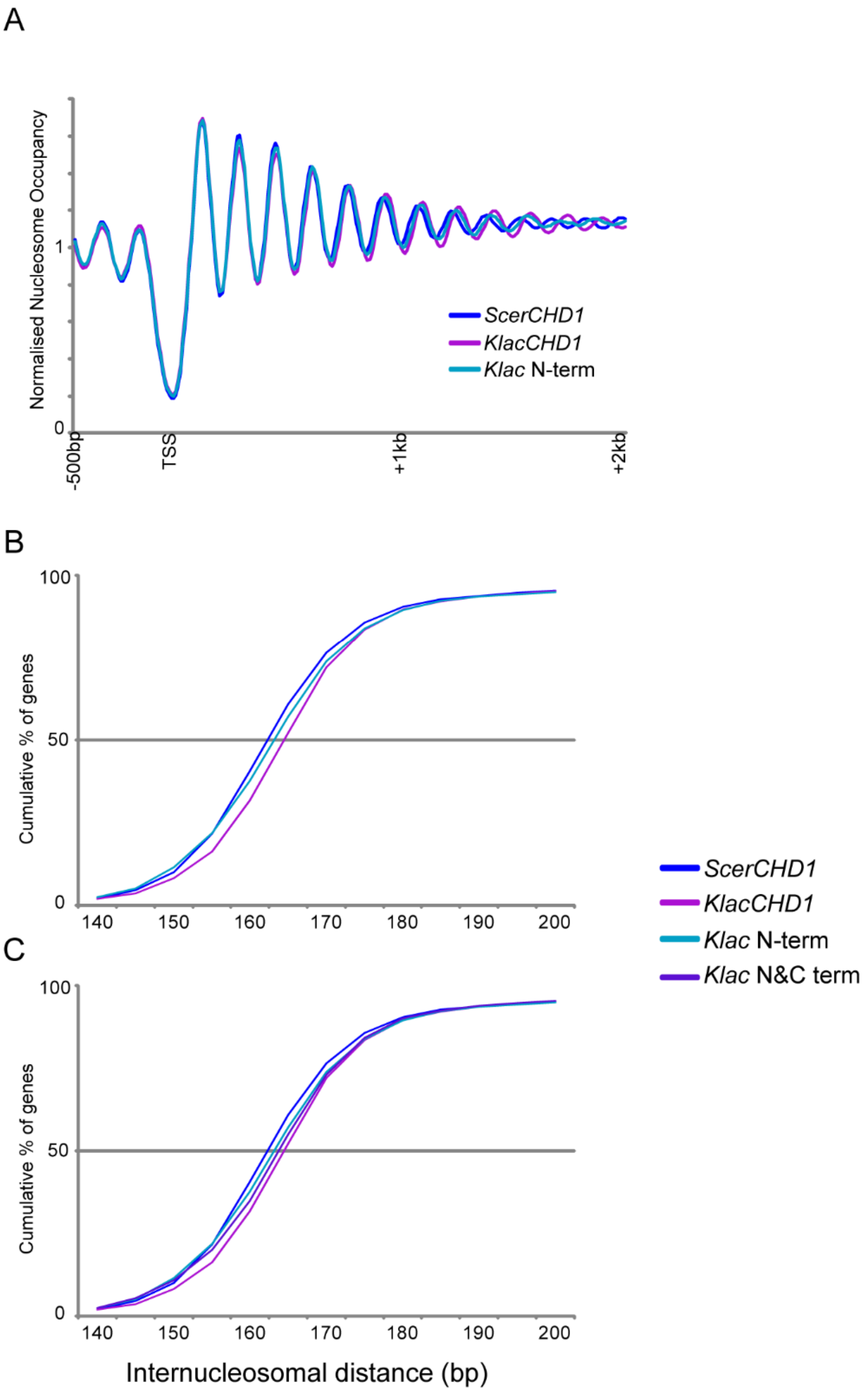

Figure S4 Differences between *S. cerevisiae* and *K. lactis* Chd1 that affect nucleosome spacing are distributed throughout the protein. (**A**) Genome-wide nucleosome mapping data are shown for strains carrying both species' *CHD1* orthologs, and for a strain carrying a chimaeric CHD1 with only the N-terminal portion of the *K. lactis* ortholog. (**B-C**) Cumulative distribution plots of internucleosome distances for the indicated chimaeric *CHD1* strains.

Figure S5

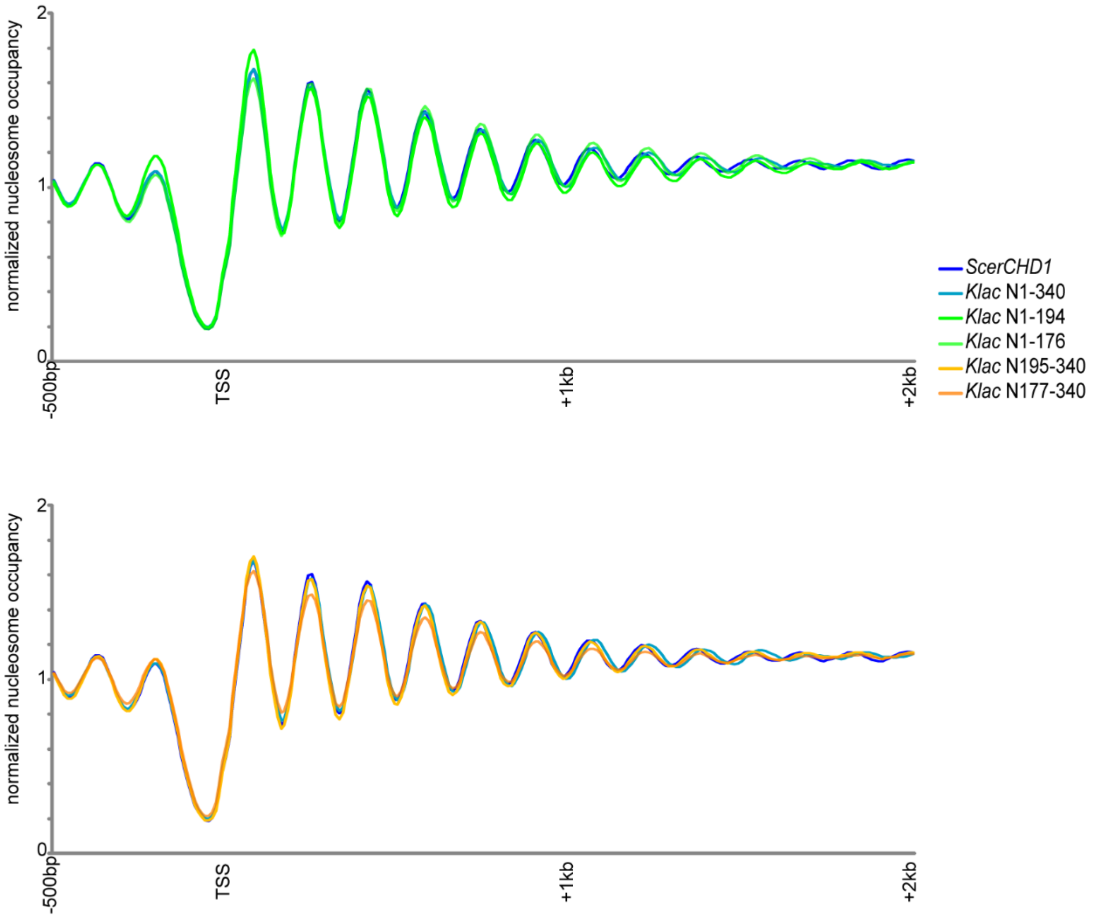

Figure S5 Genome-wide data for N-terminal swaps. Averaged TSS-aligned genome-wide nucleosome mapping data for the strains shown in **Figure 5**.

Figure S6

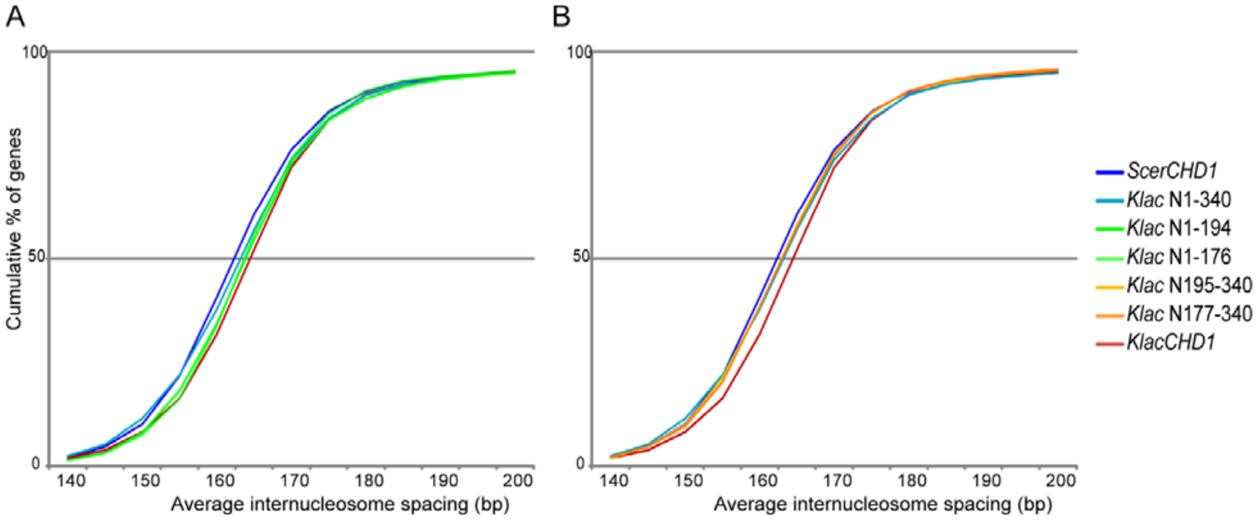

Figure S6 Distribution of nucleosome position changes in N-terminal swaps. Cumulative distribution data for internucleosome distances for Chd1 swaps affecting the indicated domains. Note the significant effect of swaps affecting the unstructured N-terminal 180 amino acids (**A**), and the lack of effect of swaps altering the chromodomains (**B**).

Figure S7

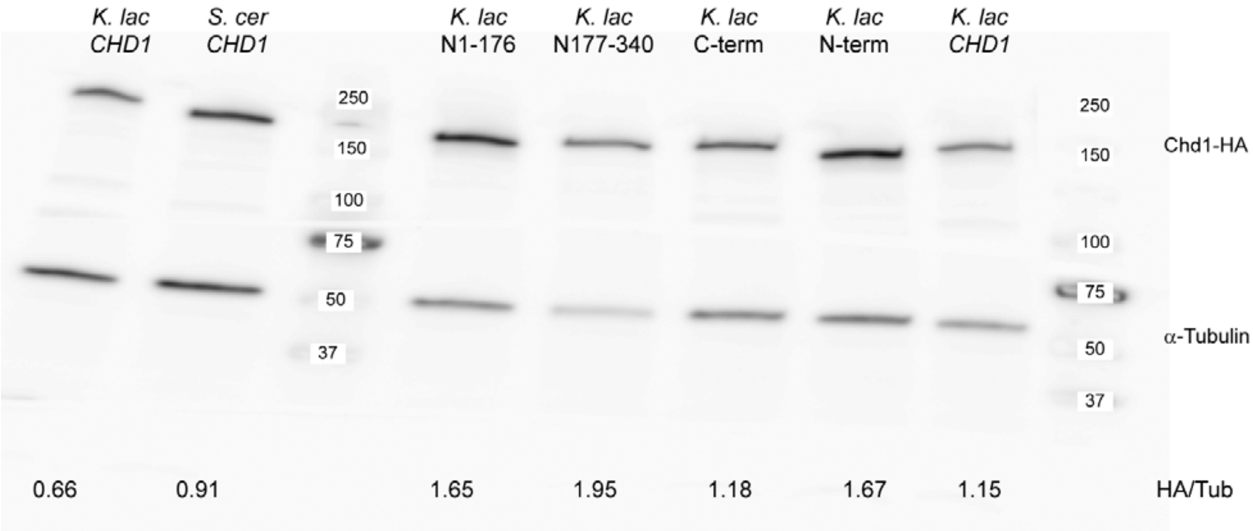

Figure S7 Chd1 abundance is unaffected by *K. lactis* sequence. Lysates of HA-tagged Chd1 strains were blotted for HA and α-tubulin (control), showing no correlation between Chd1 protein abundance and in vivo nucleosome spacing activity.

Figure S8

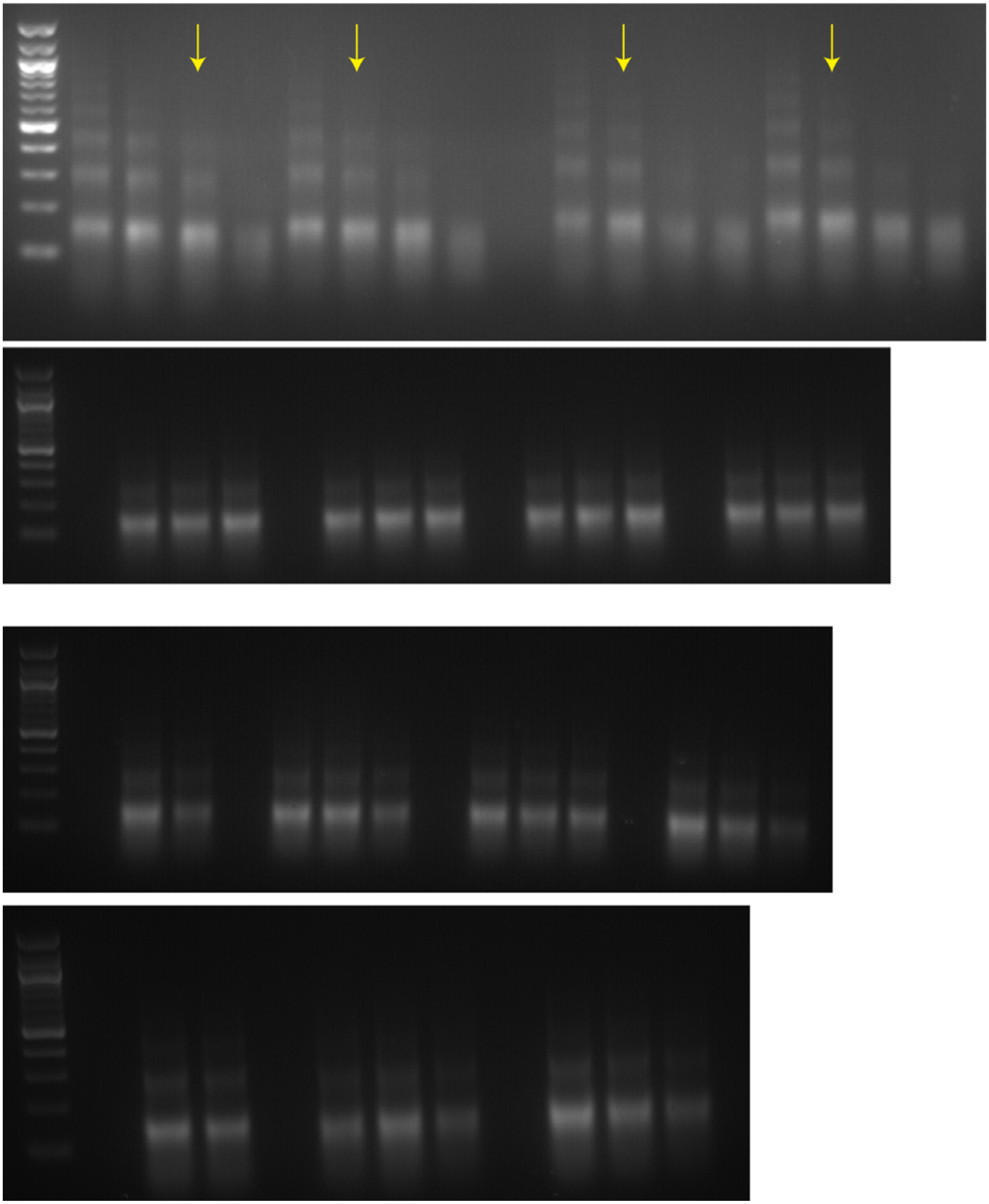

Figure S8 Example MNase digestions. Four representative experiments, with top panel showing a wide MNase titration for four strains – yellow arrows indicate the MNase level chosen for purification of mononucleosomal DNA. For the remaining three panels, MNase level was titrated more closely around a target concentration, resulting in more similar laddering for each titration step.
