# Supplementary material for: Comparative Genomics Reveals Chd1 as a Determinant of Nucleosome Spacing in Vivo
Source: G3 (Bethesda). 2015 Jul 14;5(9):1889–97. doi: 10.1534/g3.115.020271 (PMC4555225; doi:10.1534/g3.115.020271)
Supplement: Supporting Information [file supp_g3.115.020271_FigureS1.pdf]

Figure S1

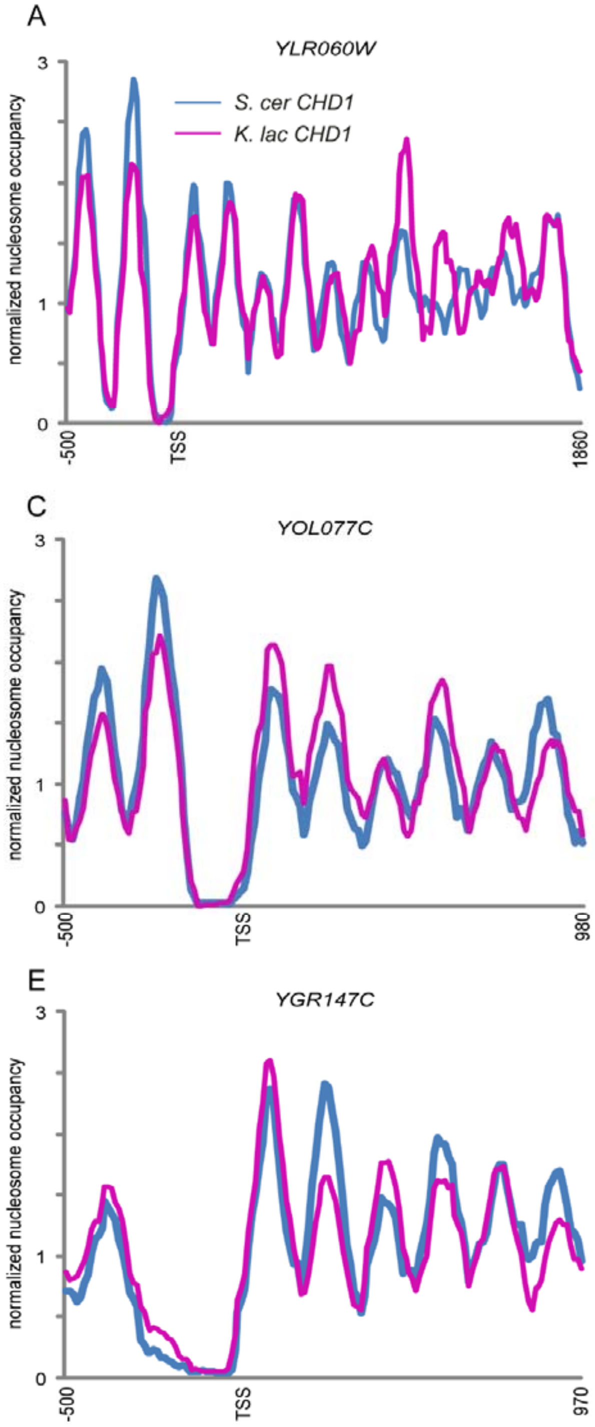

Figure S1 Individual gene examples showing effects of *K. lactis CHD1* orthologue on nucleosome positioning. Six individual genes from the dataset shown in **Figure 2**, including examples with evident nucleosome shifts as well as genes with no discernable effects of the *CHD1* swap.
