# Supplementary material for: Comparative Genomics Reveals Chd1 as a Determinant of Nucleosome Spacing in Vivo
Source: G3 (Bethesda). 2015 Jul 14;5(9):1889–97. doi: 10.1534/g3.115.020271 (PMC4555225; doi:10.1534/g3.115.020271)
Supplement: Supporting Information [file supp_g3.115.020271_FigureS2.pdf]

Figure S2

A

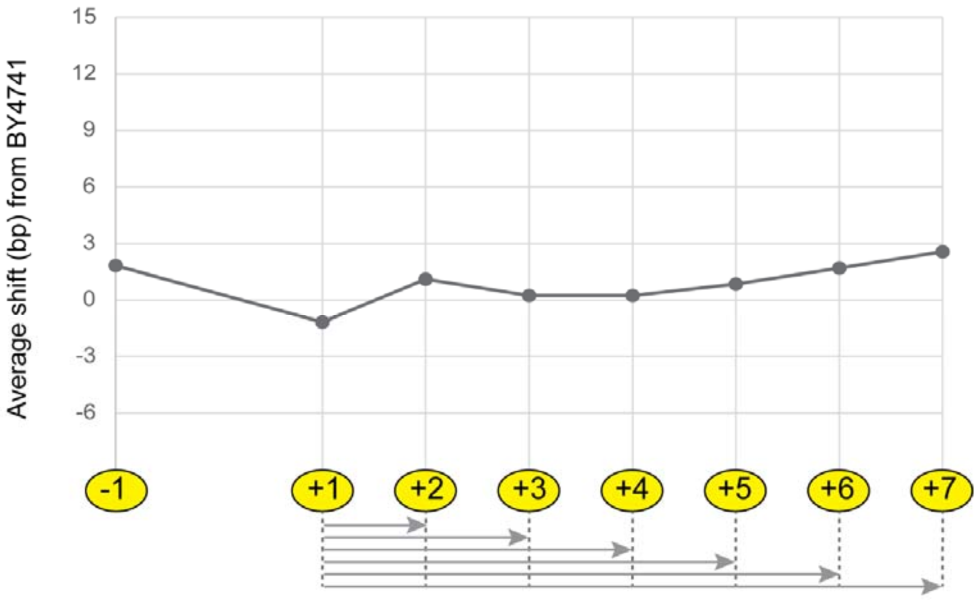

BY4741	168	334	495	655	815	971
S.cer Chd1 (integrated)	168<br>(0.25)	333<br>(0.96)	495<br>(0.52)	656<br>(0.26)	816<br>(0.31)	972<br>(0.89)

B

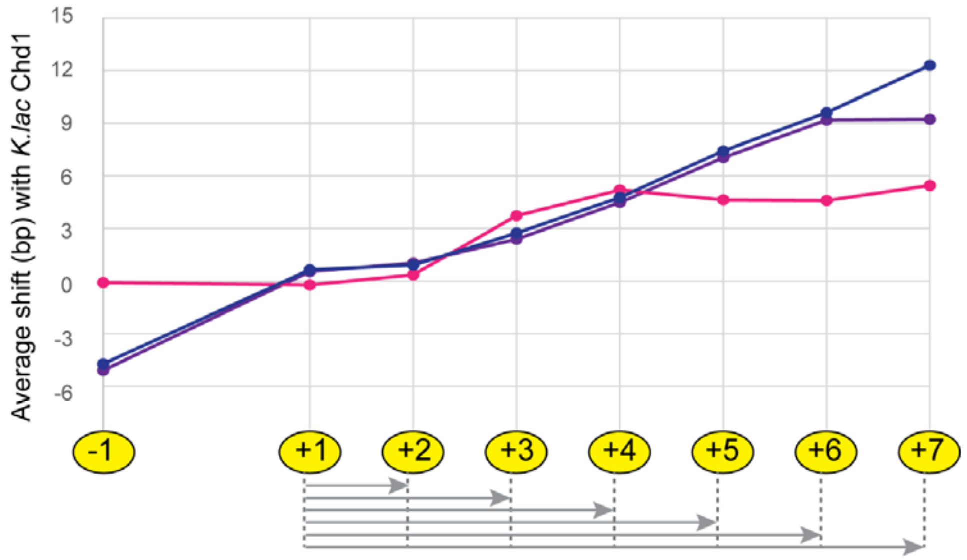

S.cer Chd1 (integrated)2	169	332	495	658	819	975
K.lac Chd1 (integrated)2	170<br>(0.069)	336<br>(8.8E-7)	502<br>(1.8E-12)	665<br>(5.8E-11)	825<br>(2.3E-8)	983<br>(1.9E-7)
S.cer Chd1 (plasmid)	170	336	501	666	828	991
K.lac Chd1 (plasmid)	175<br>(2.2E-7)	344<br>(6.3E-12)	512<br>(2.8E-15)	679<br>(1.2E-17)	843<br>(2.7E-19)	1007<br>(8.9E-15)
S.cer Chd1 (plasmid)2	172	338	505	669	833	999
K.lac Chd1 (plasmid)2	177<br>(1.1E-7)	345<br>(8.5E-14)	514<br>(3.8E-16)	682<br>(5.3E-23)	849<br>(9.0E-29)	1019<br>(4.0E-30)

Figure S2 The *K. lactis CHD1* orthologue can direct wider spacing of *S. cerevisiae* nucleosomes from both plasmid and endogenous expression. Plot of shifts in -1 to +7 nucleosome positions between *S. cerevisiae* and *K. lactis CHD1* swap strains, as in **Figure 2B-C**. In each case, top panel shows distances between equivalent nucleosomes for the pair of strains indicated, while bottom panel shows the average distance of genic nucleosomes from the +1 nucleosome, along with corresponding p-values for the swap strains. (**A**) shows that this metric is specific, as no significant changes are found between wild-type yeast and a "pseudo wild-type" with a genomic *chd1Δ* deletion covered by a plasmid-borne *CHD1* gene. (**B**) shows that effects of *K. lactis CHD1* on nucleosome spacing are reproducible both for genomically-integrated swap strains as well as plasmid-borne *CHD1* swaps.
