# Supplementary material for: Comparative Genomics Reveals Chd1 as a Determinant of Nucleosome Spacing in Vivo
Source: G3 (Bethesda). 2015 Jul 14;5(9):1889–97. doi: 10.1534/g3.115.020271 (PMC4555225; doi:10.1534/g3.115.020271)
Supplement: Supporting Information [file supp_g3.115.020271_FigureS3.pdf]

Figure S3

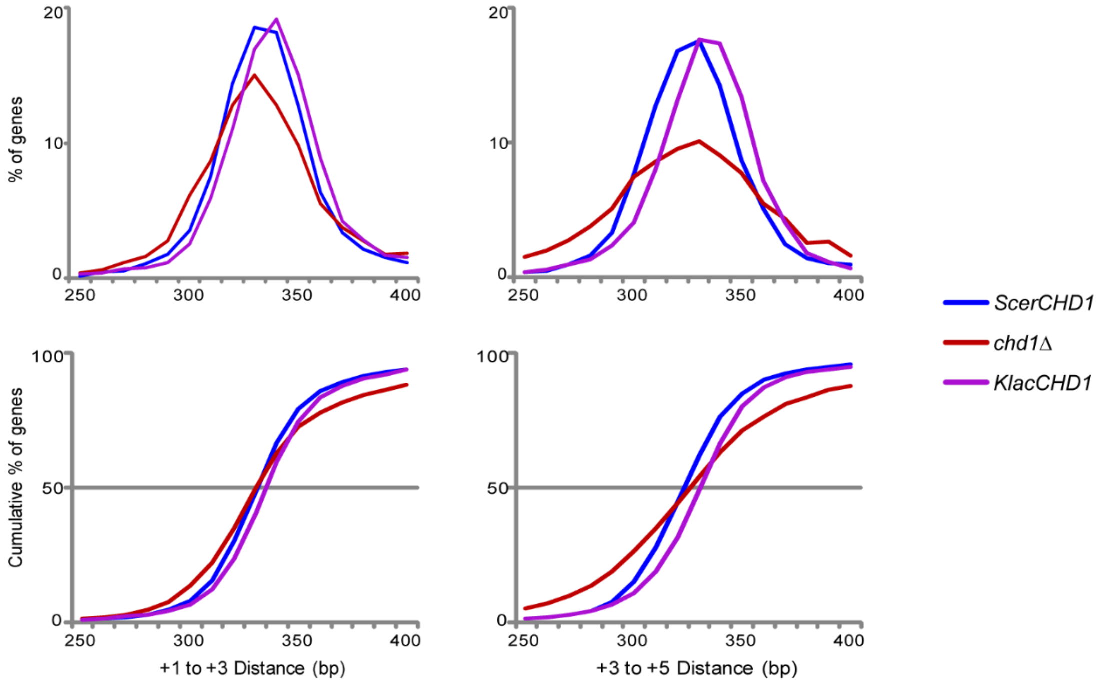

Figure S3 Distribution of nucleosome spacing changes in *CHD1* swap strains. Quantitation of internucleosome distances in the three strains detailed in **Figure 2A**. For each nucleosome mapping dataset, nucleosome positions were called as in (WEINER *et al.* 2010). Left panels show distances from all ORF +1 nucleosomes to the corresponding +3 nucleosome, while right panels show +3 to +5 nucleosome distances. Top panels show the histogram of all such distances for the three strains, color-coded as in (A), while bottom panels show the same data as cumulative distribution plots. A replicate for these integrated strains as well as 2 replicates for plasmid-borne *CHD1* strains are shown in **Supplemental Figure S2**.
