# Supplementary material for: Comparative Genomics Reveals Chd1 as a Determinant of Nucleosome Spacing in Vivo
Source: G3 (Bethesda). 2015 Jul 14;5(9):1889–97. doi: 10.1534/g3.115.020271 (PMC4555225; doi:10.1534/g3.115.020271)
Supplement: Supporting Information [file supp_g3.115.020271_FigureS4.pdf]

Figure S4

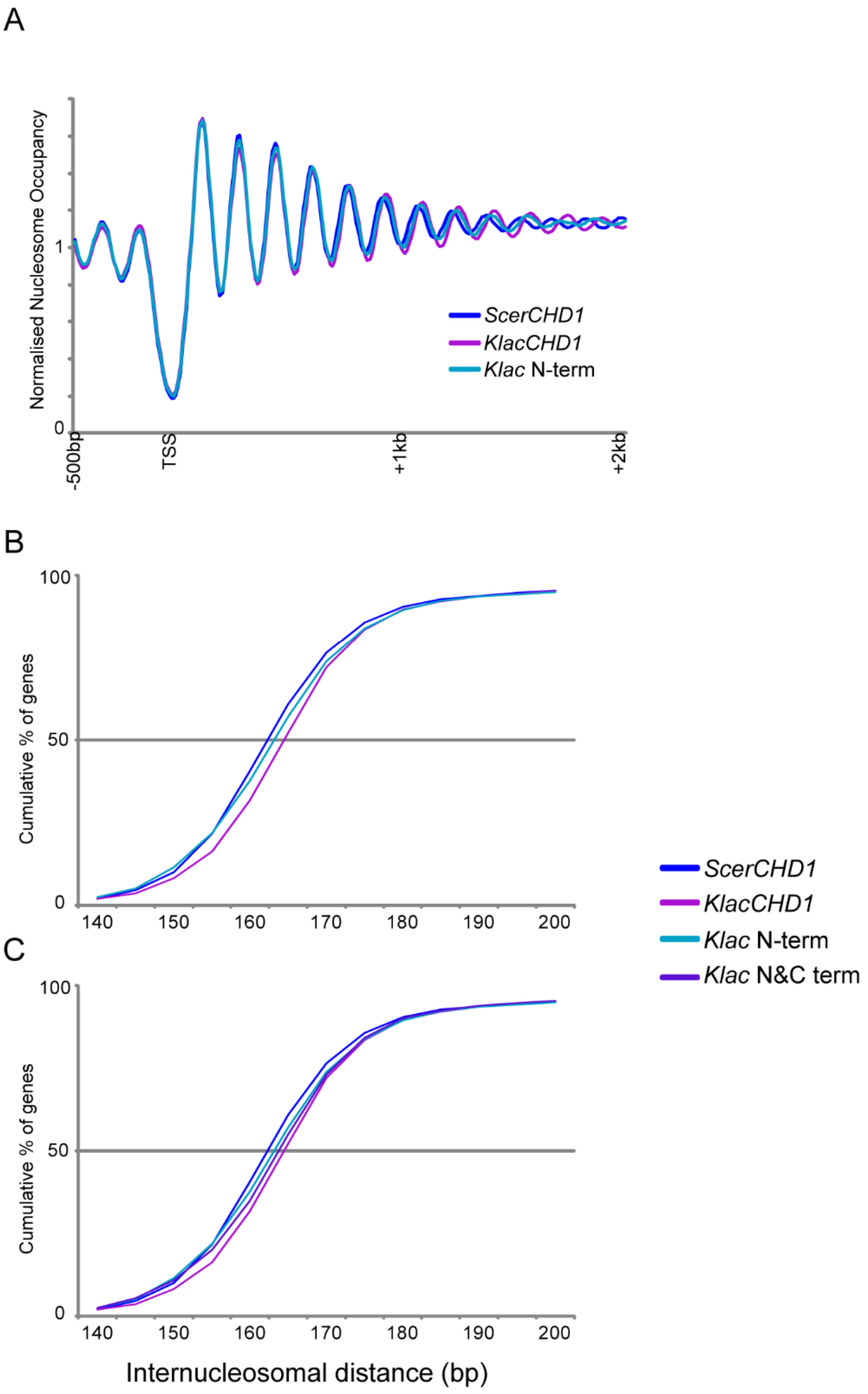

Figure S4 Differences between *S. cerevisiae* and *K. lactis* Chd1 that affect nucleosome spacing are distributed throughout the protein. (**A**) Genome-wide nucleosome mapping data are shown for strains carrying both species' *CHD1* orthologs, and for a strain carrying a chimaeric CHD1 with only the N-terminal portion of the *K. lactis* ortholog. (**B-C**) Cumulative distribution plots of internucleosome distances for the indicated chimaeric *CHD1* strains.
