# Supplementary material for: Comparative Genomics Reveals Chd1 as a Determinant of Nucleosome Spacing in Vivo
Source: G3 (Bethesda). 2015 Jul 14;5(9):1889–97. doi: 10.1534/g3.115.020271 (PMC4555225; doi:10.1534/g3.115.020271)
Supplement: Supporting Information [file supp_g3.115.020271_FigureS5.pdf]

Figure S5

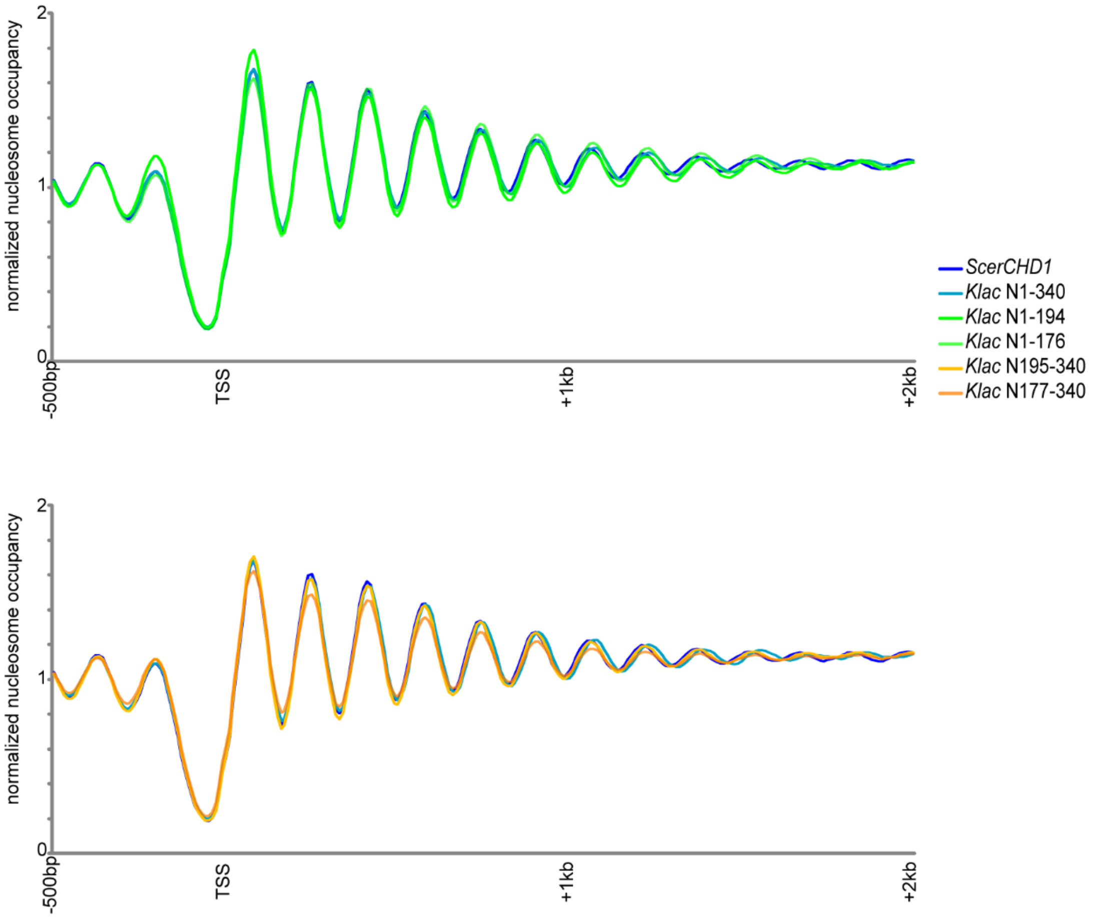

Figure S5 Genome-wide data for N-terminal swaps. Averaged TSS-aligned genome-wide nucleosome mapping data for the strains shown in **Figure 5**.
