# Supplementary material for: Comparative Genomics Reveals Chd1 as a Determinant of Nucleosome Spacing in Vivo
Source: G3 (Bethesda). 2015 Jul 14;5(9):1889–97. doi: 10.1534/g3.115.020271 (PMC4555225; doi:10.1534/g3.115.020271)
Supplement: Supporting Information [file supp_g3.115.020271_FigureS6.pdf]

Figure S6

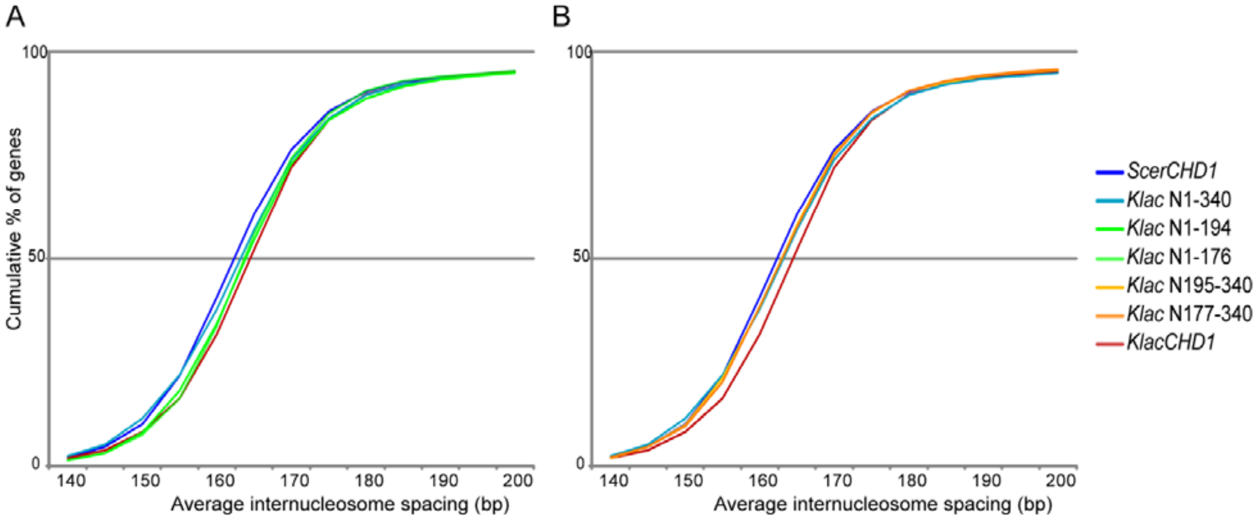

Figure S6 Distribution of nucleosome position changes in N-terminal swaps. Cumulative distribution data for internucleosome distances for Chd1 swaps affecting the indicated domains. Note the significant effect of swaps affecting the unstructured N-terminal 180 amino acids (**A**), and the lack of effect of swaps altering the chromodomains (**B**).
