# Supplementary material for: Comparative Genomics Reveals Chd1 as a Determinant of Nucleosome Spacing in Vivo
Source: G3 (Bethesda). 2015 Jul 14;5(9):1889–97. doi: 10.1534/g3.115.020271 (PMC4555225; doi:10.1534/g3.115.020271)
Supplement: Supporting Information [file supp_g3.115.020271_FigureS7.pdf]

Figure S7

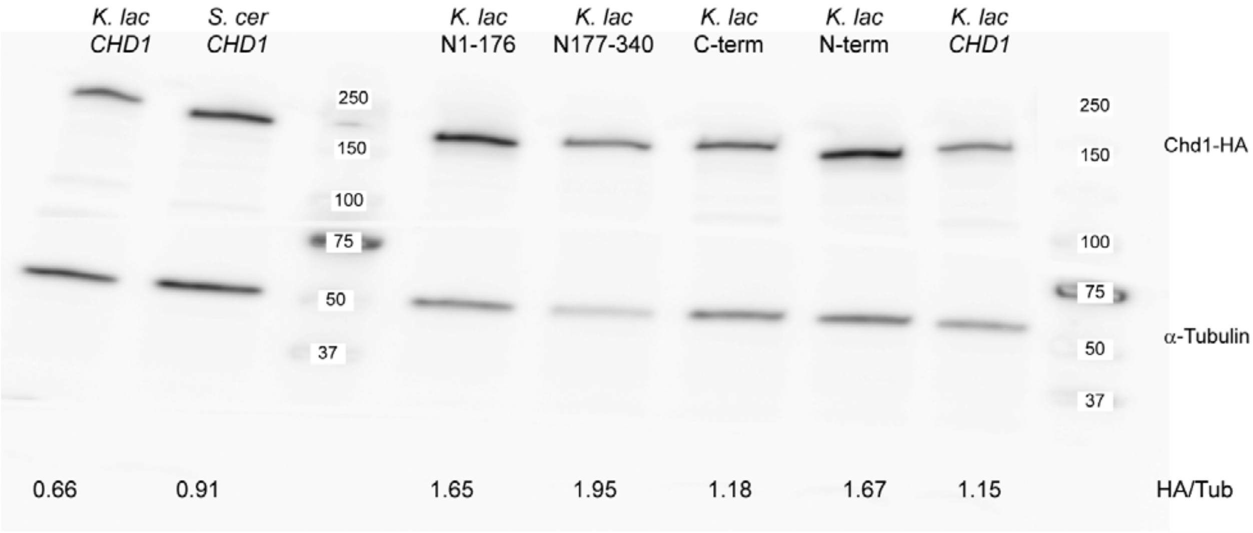

Figure S7 Chd1 abundance is unaffected by *K. lactis* sequence. Lysates of HA-tagged Chd1 strains were blotted for HA and α-tubulin (control), showing no correlation between Chd1 protein abundance and in vivo nucleosome spacing activity.
