# Supplementary material for: Comparative Genomics Reveals Chd1 as a Determinant of Nucleosome Spacing in Vivo
Source: G3 (Bethesda). 2015 Jul 14;5(9):1889–97. doi: 10.1534/g3.115.020271 (PMC4555225; doi:10.1534/g3.115.020271)
Supplement: Supporting Information [file supp_g3.115.020271_FigureS8.pdf]

Figure S8

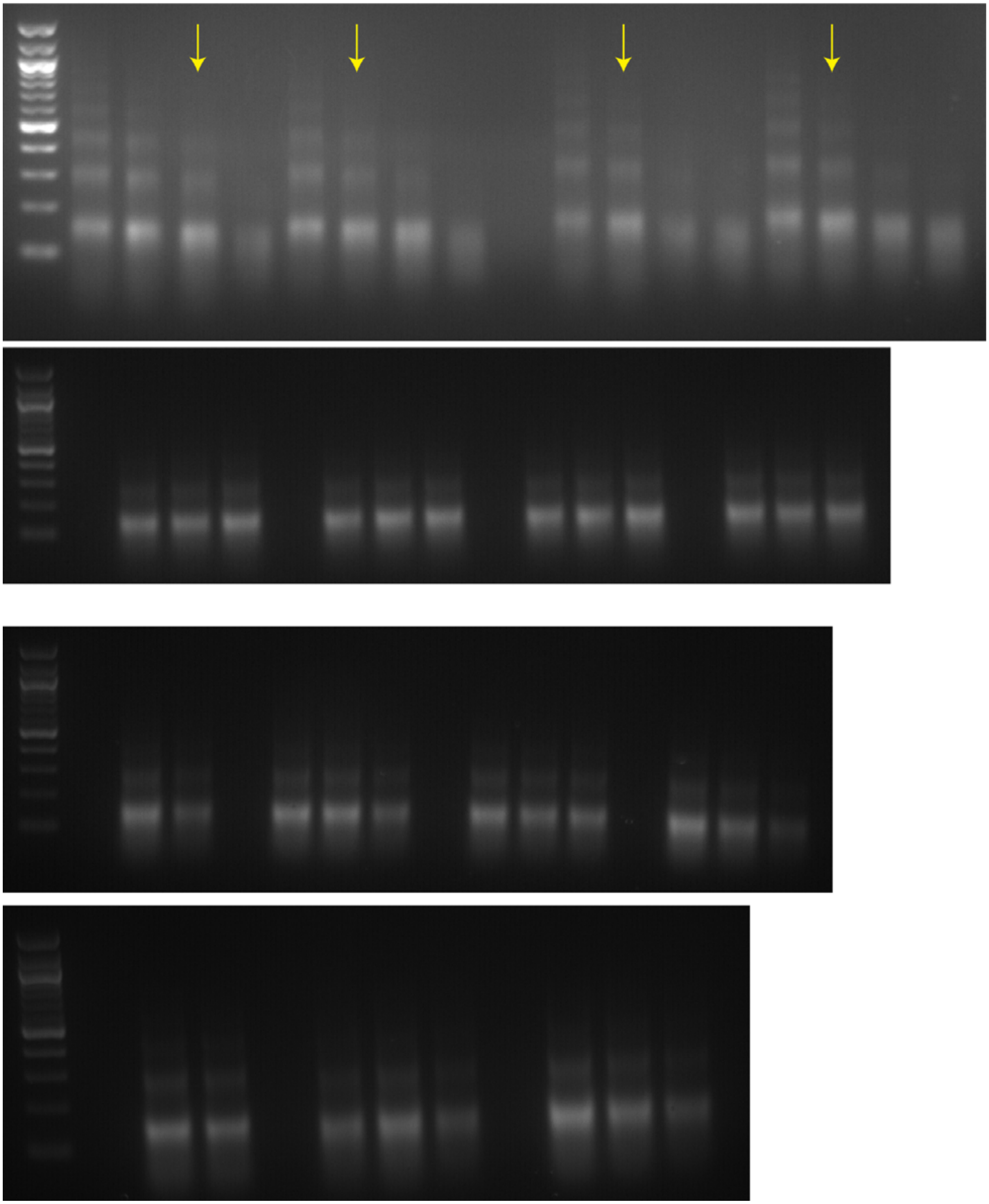

Figure S8 Example MNase digestions. Four representative experiments, with top panel showing a wide MNase titration for four strains – yellow arrows indicate the MNase level chosen for purification of mononucleosomal DNA. For the remaining three panels, MNase level was titrated more closely around a target concentration, resulting in more similar laddering for each titration step.
